# EEG-Based Machine Learning Classification of Shooting Conditions and Performance Levels During the Shooting Preparation Stage

**DOI:** 10.3390/brainsci16101014

**Published:** 2026-09-24

**Authors:** Ting Shi, Yueping Peng, Xiuyan Hu, Xinyu Shi, Yunfa Fu, Anmin Gong

**Affiliations:** 1 School of Information Engineering, Engineering University of the Chinese People’s Armed Police Force (PAP), Xi’an 710086, China; lovestforever1209@163.com (T.S.); percy001@163.com (Y.P.); huxiuyan2024@163.com (X.H.); 18729090681@163.com (X.S.); 2 Faculty of Information Engineering and Automation, Kunming University of Science and Technology, Kunming 650500, China; 3 School of Life Science and Technology, Xi’an Jiaotong University, Xi’an 710049, China

**Keywords:** electroencephalogram (EEG), machine learning (ML), shooting sports, feature extraction, classification

## Abstract

**Highlights:**

**What are the main findings?**

- Pre-shot EEG features distinguished normal, noise, and low-light shooting conditions and also separated high- and low-performance trials within the same condition.
- FBCSP combined with SVM showed the strongest overall decoding performance, while beta-band features showed relatively consistent discriminative contributions across classification tasks.

**What are the implications of the main findings?**

- EEG-based ML provides preliminary evidence that pre-shot EEG features contain discriminative information across sensory conditions and behaviorally defined performance groups. The present results do not establish a deployable monitoring method; larger subject-independent studies are required.
- Features preserving spatial patterns and functional connectivity may be more suitable than highly compressed global indices for decoding performance-related EEG activity in shooting.

**Abstract:**

**Background:** Shooting performance depends on environmental conditions and neural state during preparation. Few studies have applied machine learning (ML) to classify shooting conditions and performance levels using EEG. This study assessed whether pre-shot EEG features could discriminate shooting conditions and performance levels within the same condition. **Methods:** EEG and behavioral data from 35 shooters were analyzed under normal, noise, and low-light conditions. Band-power, phase-locking value (PLV)-based functional-connectivity, and filter bank common spatial pattern (FBCSP) features were evaluated separately using support vector machine (SVM) and random forest (RF). EEGNet served as an end-to-end benchmark for performance classification. Repeated trial-wise five-fold cross-validation assessed within-dataset trial-level discrimination, while supplementary leave-one-subject-out cross-validation (LOSO-CV) assessed generalization to unseen shooters in the performance task. **Results:** All three feature types provided discriminative information for shooting conditions and high- versus low-performance trials. FBCSP combined with SVM achieved the best within-dataset performance, reaching 91% for normal versus low-light classification and 81% for high versus low performance under the normal condition. Because trials from the same participants could occur in both training and test folds, these accuracies were not estimates of subject-independent performance. LOSO-CV accuracies ranged from 59% to 70%, indicating limited cross-subject generalization. Beta-band features showed relatively consistent discriminative contributions. Gamma-band and frontal-channel findings require caution because residual muscle activity and attenuation of frontal EEG during virtual-EOG-based correction could not be excluded. **Conclusions:** Pre-shot EEG features showed preliminary within-dataset discriminability, but inter-individual variability limited generalization. Larger independent datasets, dedicated EOG/EMG recordings, and subject-adaptation strategies are required before practical deployment.

## 1. Introduction

Shooting is a complex sport that requires a high degree of coordination between skill and psychological control. It not only demands advanced motor execution but also depends on stable mental states. Psychological factors have a substantial influence on shooting behavior. On the one hand, environmental conditions can significantly affect shooters’ psychological states; under different shooting conditions, such as lighting, noise, physiological load, and pressure, shooters may exhibit marked differences in mental state [1]. On the other hand, among experienced shooters, psychological variables such as attentional focus, emotional state, and psychological stress can directly affect shooting performance [2]. Therefore, monitoring and analyzing psychophysiological activity during the shooting preparation stage is of great importance.

Electroencephalography (EEG) is a neurophysiological signal that directly reflects brain activity and has therefore become an important tool for monitoring psychophysiological changes in shooting. Through EEG measurement, previous studies have identified characteristic changes in shooting-preparation EEG under different conditions and performance levels. A systematic review by Gong et al. [3] further showed that differences in external conditions and shooting environments can significantly modulate neural activity patterns during shooting. Specifically, Woo et al. [4] examined the effect of stress on cortico-cortical communication and found decreased connectivity between Cz and T4 and increased connectivity between Cz and T3 under stress. Hatfield et al. [5] studied EEG features under competitive shooting environments and reported increased anxiety, heart rate, and cortisol levels, together with upper-alpha desynchronization and increased cortico-cortical communication between motor and non-motor regions. Li et al. [6] investigated the neural activity of novice shooters under competitive conditions and found sex-related differences in power spectral density across theta, alpha, alpha-2, beta, and gamma bands, as well as in network features derived from phase-locking value (PLV). Gallicchio et al. [7] examined the effects of cardiovascular load on pre-shot EEG activity and found that frontal midline theta power decreased overall under load, while alpha power increased in temporal and occipital regions but not in central regions. Gu et al. [8] compared brain functional network connectivity during shooting preparation under normal, noisy, and low-light conditions and found that audiovisual interference reduced the importance of cortical regions related to audiovisual function in the whole-brain network relative to the normal condition. Man et al. [9] examined microstate features under audiovisually constrained conditions, identified task-related microstate templates, and showed that microstate characteristics differed significantly across shooting conditions. Gong et al. [10] proposed a computational brain network analysis model based on independent component analysis (ICA) and source-space clustering. By repeatedly applying ICA decomposition and clustering, together with standardized low-resolution brain electromagnetic tomography (sLORETA) for source localization, they constructed source-level brain networks and substantially improved spatial resolution.

Regarding the relationship between shooting performance and EEG, Loze et al. [11] examined the association between pre-shot alpha-band power and shooting performance and found that occipital alpha power increased before the best shots and decreased before the worst shots, suggesting that suppression of visual attention plays a key role in shooting success. Del Percio et al. [12] compared EEG activity during the shooting preparation stage and found that shooters with better performance exhibited higher event-related coherence in the alpha band across the cerebral cortex, especially between parietal visuospatial regions and other posterior cortical regions (temporal and parietal areas). They also reported a significant positive correlation between shooting scores and alpha-band event-related coherence. Gong et al. [13] found that EEG features in the beta1 and beta2 bands showed the strongest relationship with shooting performance, and that brain network features were significantly associated with shooting performance. Badarin et al. [1] reported that, during shooting, whole-brain mean delta-band power and right temporal alpha-band power increased during missed trials, while frontal and central theta-band power was negatively correlated with performance. Kuo et al. [14] examined the temporal dynamics of theta, alpha, and beta oscillations during goal-directed aiming in novice shooters and showed that alpha and beta oscillations were important for predicting aiming accuracy. Cheng et al. [15] found that superior shooting performance was characterized by stronger sensorimotor rhythms during aiming and lower coherence in the Fz-T3 region during precise shooting. Chen et al. [16] also reported that shooting precision, stability, and reaction speed were significantly associated with enhanced SMR, suppressed theta activity, and modulation of high-beta activity; the higher the integrated oscillatory modulation index, the better the shooting performance.

Previous studies have identified numerous EEG-based neural markers of brain states under different conditions and performance levels. These markers are important for understanding underlying neural mechanisms. However, when the goal is practical monitoring and guidance in real shooting training, two challenges remain. First, monitoring based on a single predefined EEG marker, such as frontal-midline theta power, may not capture the complex spectral, spatial, and connectivity-related neural information involved in shooting preparation. Second, electrophysiological characteristics vary substantially across individuals, which may limit the generalizability of EEG-based monitoring to previously unseen shooters.

Machine learning (ML) is a core technology in artificial intelligence and an important tool for applying neural markers in brain–computer interfaces and neurofeedback systems. Its outputs, such as accuracy, recall, specificity, and AUC, provide quantitative and objective evaluation. ML has been widely used in disease diagnosis, brain state recognition, and emotion monitoring. For example, Huang et al. [17] developed a pain-level classification model by combining common spatial pattern and multiple linear regression, enabling binary classification and continuous intensity prediction from single-trial laser-evoked potentials. Subasi et al. [18] used a hybrid genetic algorithm and particle swarm optimization model to optimize support vector machine parameters for classifying EEG signals and predicting epileptic seizures. Zhang et al. [19] used support vector machine and random forest methods to study the relationship between pre-anesthetic brain functional network connectivity and individual sensitivity, with the aim of predicting sensitivity to propofol. In addition, ML has enabled automated diagnosis of EEG-based epilepsy [20], Parkinson’s disease [21], Alzheimer’s disease [22], and general EEG pathology [23].

Although ML has made remarkable progress in medical applications and general brain–computer interface development, existing shooting EEG studies remain largely limited to statistical analyses of individual neural markers. To our knowledge, few studies have systematically applied ML classification models to decode both shooting conditions and performance levels. The limited adoption of ML in this field may reflect several practical challenges, including relatively small sample sizes, difficulties in synchronously recording high-quality EEG and detailed behavioral data during realistic shooting tasks, substantial inter-individual variability, heterogeneous experimental protocols, and the lack of standardized performance labels.

To address the limitation of relying on a single predefined EEG marker, the present study separately extracted and evaluated the following three complementary EEG feature representations: band power, PLV-based functional connectivity, and FBCSP spatial features. Their classification performance was compared to examine the relative discriminative value of spectral, connectivity, and spatial information for shooting-condition and shooting-performance classification. To examine the influence of inter-individual variability, trial-wise five-fold cross-validation was used as the primary evaluation of within-dataset trial-level discriminability, whereas LOSO-CV was additionally applied to the shooting-performance classification task as a supplementary subject-independent evaluation. LOSO-CV was intended to quantify, rather than eliminate, the effect of inter-individual variability on generalization to previously unseen shooters. Accordingly, the present study should be interpreted as a preliminary evaluation of EEG discriminability rather than as validation of a deployable monitoring system.

## 2. Materials and Methods

### 2.1. Experimental Data

The data used in this study were derived from the shooting behavior and synchronized EEG dataset reported in brain network research of skilled shooters in the shooting preparation stage under the condition of limited sensory function. The original experimental design employed a priori power analysis using G*Power 3.1 [24] to ensure adequate statistical power. Based on a repeated-measure analysis of variance (ANOVA) model, the parameters were set as follows: medium effect size (f = 0.25), significance level (α = 0.05), and target power of 0.80. The final sample of 35 participants met the preset power requirement and provided a reliable data foundation for exploratory EEG feature analysis and ML modeling in shooting.

The experimental conditions and procedure are shown in Figure 1a. A total of 35 participants performed standing pistol shooting at a chest ring target under the following three conditions: normal, noise, and low light. Each condition contained two blocks of 30 trials, yielding 180 trials per participant. Shooting behavior data were recorded by an optical shooting training system and included the following seven indices: horizontal aiming deviation (SX), vertical aiming deviation (SY), center of gravity of the aiming curve (COG), aiming time index (ATI), relative trigger value (RTV), trigger integrated control efficiency (TIRE), and shooting score (SCORE); detailed definitions are provided in Table 1. The behavioral data were structured as a 35 × 60 × 7 matrix, representing 35 participants, 60 trials, and 7 behavioral indices. EEG signals were simultaneously acquired using a Neuracle NSW332 32-channel wireless amplifier (Neuracle Technology Co., Ltd., Shanghai, China). Electrode placement followed the international 10–20 system (see Figure 1a), with bilateral mastoids serving as the reference electrodes.

### 2.2. Data Analysis Process

The overall data acquisition and analysis workflow is illustrated in Figure 1. Figure 1a depicts the experimental acquisition procedure, in which shooting was performed under normal, noise, and low-light conditions, while EEG signals and behavioral indices were recorded synchronously. Figure 1b outlines the preprocessing procedure, including artifact rejection, clustering, sample labeling, reference electrode removal, and frequency-band decomposition. Figure 1c presents EEG feature extraction and ML modeling, including band power, PLV-based functional connectivity, and FBCSP features, which were subsequently used to train the ML models. Figure 1d illustrates the performance evaluation of the trained models, based on classification accuracy, specificity, recall, the receiver operating characteristic (ROC) curve, and feature importance analysis.

### 2.3. Experimental Design and Sample Partitioning

This study included two classification experiments based on pre-shot EEG signals. Experiment 1 examined the discriminability of shooting conditions by classifying EEG trials recorded under normal, noise, and low-light conditions. Pairwise comparisons were conducted for normal versus noise, normal versus low light, and noise versus low-light conditions.

Experiment 2 examined the discriminability of shooting-performance levels within each shooting condition. The seven behavioral indices were first used to construct high-, medium-, and low-performance clusters. For the primary binary classification analysis, only the high- and low-performance clusters were retained, and their corresponding EEG trials were classified separately under each condition. The medium-performance cluster was excluded from the primary binary analysis because it showed greater behavioral overlap; this design was intended to examine EEG discriminability between clearly distinct performance groups and does not represent a full-spectrum or three-class classification task.

For both experiments, band-power, PLV-based functional-connectivity, and FBCSP features were extracted and evaluated separately. Each handcrafted feature set was independently classified using SVM and RF, whereas EEGNet was used as an end-to-end comparison model. Repeated trial-wise five-fold cross-validation was used as the primary evaluation for both experiments to assess within-dataset trial-level discriminability. For Experiment 2, LOSO-CV was additionally conducted as a supplementary evaluation of subject-independent shooting-performance classification.

To facilitate ML analysis, the experimental samples were then partitioned and labeled. In Experiment 1, conditional labels (normal, noise, and low light) were directly assigned to the EEG data. Experiment 2 aimed to classify EEG signals corresponding to different shooting-performance levels within the same condition.

For Experiment 2, trials with abnormal behavioral indices were first excluded. K-means clustering was then applied to the seven behavioral indices of each trial to divide shooting performance into three clusters. To define the performance levels in a data-driven and objective manner, the cluster centroid with the lowest mean shooting score was designated as the low-performance class. In the multidimensional behavioral feature space, the Euclidean distances between the remaining two cluster centroids and the low-performance centroid were calculated, and the cluster farthest from the low-performance centroid was defined as the high-performance class, with the remaining cluster defined as the medium-performance class. This clustering strategy was reviewed by expert pistol-shooting instructors and was broadly consistent with their judgments of overall shooting performance. For the binary classification analysis, only the high- and low-performance clusters were retained, whereas the medium-performance cluster was excluded. To quantify label stability, silhouette coefficients were computed for the selected behavioral data. In addition, t-distributed stochastic neighbor embedding (t-SNE) [25] was used for visualization, and a variance-contribution approach was applied to assess the influence of each behavioral index on the clustering results.

Given the limited number of trials available for each participant and condition in this exploratory study, the primary analysis pooled trials across participants and treated each trial as an independent observation. This trial-level design was selected to evaluate whether pre-shot EEG features contained discriminative information for shooting-condition and shooting-performance classification within the present dataset, while preserving trial-to-trial variability. Similar pooled-trial classification strategies have been used in EEG-based affective-state research [26,27].

However, because trials from the same participant could occur in both the training and test folds, repeated trial-wise five-fold cross-validation was interpreted as an estimate of within-dataset trial-level discrimination rather than subject-independent generalizability. Therefore, for the shooting-performance classification task, LOSO-CV was additionally performed to assess classification performance in previously unseen participants and to quantify the influence of inter-individual variability.

### 2.4. EEG Preprocessing

Raw EEG signals were recorded at 1000 Hz and contained neural activity as well as ocular artifacts, environmental noise, and other physiological artifacts. The preprocessing pipeline is shown in Figure 1b. First, the continuous data were downsampled to 250 Hz. Second, a 50 Hz notch filter was applied to reduce power-line interference. Third, the signals were band-pass filtered between 4 and 70 Hz using a 100th-order FIR filter with a Hamming window, implemented in zero-phase forward-and-reverse mode (filtfilt). The shot onset of each trial was defined as time 0, and the 3 s preceding shot onset were extracted for subsequent analysis, yielding 750 samples per channel per trial.

Before subsequent analysis, EEG recordings were inspected to identify datasets with obvious bad channels or severe recording artifacts. Unusable recordings were excluded, and the remaining usable trials were retained for subsequent analysis.

Ocular-artifact correction was performed using FastICA separately for each trial. To improve decomposition stability, PCA was first applied to the whitened data and the dimensionality was reduced to 15 components before ICA. Because dedicated EOG electrodes were not available, Fp1 and Fp2 were used as virtual frontal references to identify putative ocular-artifact components. Components showing an absolute Pearson correlation coefficient greater than 0.5 with the Fp1/Fp2 reference signals were identified as putative ocular components and removed before signal reconstruction. This procedure was intended to reduce ocular contamination; however, because Fp1/Fp2 may also contain genuine frontal cortical activity, it may not completely distinguish ocular artifacts from frontal neural signals.

The two mastoid reference electrodes were removed, leaving 30 EEG channels for subsequent analysis. After preprocessing, the final numbers of valid trials retained under the normal, noise, and low-light conditions were 1740, 1620, and 1680, respectively. EEG frequency bands were defined as theta (4–8 Hz), alpha (8–12 Hz), beta (12–30 Hz), and gamma (30–70 Hz).

Because dedicated EMG channels were not recorded, residual facial, jaw, neck, or postural muscle activity, particularly in the gamma band, could not be fully excluded. Gamma-band results were therefore interpreted as discriminative signal features rather than unequivocal cortical neural activity.

### 2.5. EEG Feature Extraction

To address the non-stationary nature of EEG signals during the shooting preparation stage, the following three types of features were extracted in this study: band power, PLV-based functional connectivity, and FBCSP features, so as to decode shooting-related brain states from different dimensions. After extracting these handcrafted features, they were separately input into two classical ML models, SVM and RF, to examine the classification performance of different feature sets for shooting EEG. In addition, a lightweight deep-learning model, EEGNet, was also introduced to provide a more comprehensive evaluation of classification performance. Unlike the handcrafted-feature pipeline described above, EEGNet adopts an end-to-end learning strategy, directly inputting the preprocessed EEG time series for automatic feature extraction and classification. The corresponding comparison is presented in detail in the Appendix A.

#### 2.5.1. Frequency Band Power

Band power features are easy to extract and have clear physiological meaning. They reflect EEG activity in different frequency bands and are among the most widely used basic features in EEG research. For each EEG channel, the band power of theta, alpha, beta, and gamma bands was calculated using the band feature function in MATLAB(R2024b) with the EEG Feature Extraction Toolbox [28]. In total, 120 band power features were obtained from the 30 EEG channels.

#### 2.5.2. PLV Brain Functional Connectivity Features

Functional connectivity features capture the coordination and complex dynamic relationships between brain regions and provide a more comprehensive characterization of spatial EEG patterns [29]. In this study, PLV was used to quantify functional connectivity strength. This method measures the degree of coupling by calculating phase synchronization between signals and is a simple and widely used connectivity measure [30,31]. The PLV calculation procedure was as follows:

(1) A 100th-order finite impulse response (FIR) filter was used to band-pass filter the EEG signals, yielding frequency-specific signals.
(2) The filtered EEG signals were subjected to Hilbert transform to obtain the analytic signal:
  (1) $${\tilde{S}}_{x}\left(t\right)=\frac{1}{\pi }\int_{-\infty }^{+\infty }\frac{S_{x}(t)}{t-\tau }d\tau$$
  where $s_{x}(t)$ represents the original EEG signal, and ${\tilde{S}}_{x}\left(t\right)$ denotes the signal after Hilbert transform.
(3) Based on the original signal and its conjugate signal, the phase of each signal was calculated:
  (2) $$\varphi_{x}\left(t\right)=arctan\frac{{\tilde{S}}_{x}\left(t\right)}{S_{x}(t)}$$
  where $\varphi_{x}(t)$ is the phase value of the signal $s_{x}(t)$.
(4) Based on the phase values of the two EEG signals, the instantaneous phase difference at each sample point was computed, and the PLV between two channels within the time window was then obtained.
  (3) $$PLV=\frac{1}{N}\left|\sum_{n=1}^{N}e^{i∆\varphi (t_{n})}\right|$$
  where PLV represents the phase locking value between two electrodes within the time window, and N denotes the number of signal sampling points in that window.

When using PLV-based functional connectivity features for ML, all connections were retained. The lower triangular elements of the PLV matrix were flattened column-wise into a feature vector, yielding 435 features per frequency band and 1740 features across the four bands. Because this number greatly exceeded the sample size, feature selection was performed using Elastic Net regularization (α = 0.5) [32] to obtain a feature vector of appropriate length.

#### 2.5.3. FBCSP Algorithm

The FBCSP algorithm [33,34,35] is an improved method based on common spatial pattern (CSP) [36] and is specifically designed to extract features from the spatial distribution of EEG signals. Its core idea is to divide a broad EEG frequency range into multiple sub-bands and apply CSP to each sub-band separately, thereby extracting more discriminative features. The basic implementation procedure of FBCSP was as follows:

(1) Frequency-band partitioning: the 4–40 Hz range was divided into nine sub-bands with a step size of 4 Hz.
(2) CSP-based feature extraction was performed for each sub-band:
  (1) the average covariance matrices of the two classes were calculated;
  (2) the mixed covariance matrix was whitened;
  (3) principal component decomposition was applied to the whitened covariance matrix to obtain the feature matrix;
  (4) the first m columns and the last m columns of the feature matrix were selected to construct the spatial filter, and the most discriminative feature vectors, with a total of 2 m features, were extracted.
(3) For each sub-band, the extracted CSP features were selected and only the most discriminative ones were retained.
(4) The features from multiple sub-bands were fused to form the final feature vector.

After comparison, m was set to 10. Thus, each trial generated 20 features, and a total of 180 features were obtained across the nine sub-bands.

### 2.6. Classification Methods and Model Construction

Unless otherwise stated, classifier hyperparameters were fixed before the final cross-validation analyses and were kept identical across classification tasks. Hyperparameters were not selected using the held-out test fold.

This study employed support vector machine (SVM), random forest (RF), and EEGNet to classify EEG signals and to compare the performance of conventional ML methods and end-to-end deep learning under different feature representations. SVM and RF were used for handcrafted feature classification, whereas EEGNet was used as an end-to-end benchmark. To ensure consistency across models, all classification tasks were performed under the same data partitioning and cross-validation framework.

#### 2.6.1. SVM

SVM is a supervised learning algorithm based on statistical learning theory, and its core idea is to construct a maximum-margin hyperplane for class separation [37]. Because EEG features are typically high-dimensional, nonlinear, and characterized by complex class boundaries, a radial basis function (RBF) kernel was adopted in this study to map the original features into a higher-dimensional space and enhance separability [38]. The penalty parameter was set to 1, and the kernel scale parameter was set to auto. To reduce the influence of feature-scale differences on classification results, all input features were Z-score standardized within each training fold. No further SVM hyperparameter search was performed. The same RBF-kernel configuration was used for all handcrafted feature sets and classification tasks.

#### 2.6.2. RF

RF is an ensemble learning-based classification method that improves model stability and robustness by constructing multiple decision trees and aggregating their predictions [39]. Its core mechanism includes bootstrap sampling and random feature subset selection at each node split, which helps reduce dependence on any single tree and mitigates overfitting on high-dimensional EEG features. Under the experimental settings of this study, the number of trees was set to 100. Each tree was built using templateTree, with the minimum leaf size set to 1. The final classification result was determined by majority voting. At each split, the predictor subset was randomly sampled according to the default MATLAB random-forest setting. No additional hyperparameter search was performed; the number of trees and minimum leaf size were fixed at 100 and 1, respectively, for all analyses.

#### 2.6.3. EEGNet

To compare deep learning with conventional ML in EEG classification tasks, EEGNet was introduced in this study. EEGNet is a lightweight convolutional neural network designed for EEG signals and can perform end-to-end spatiotemporal feature extraction [40]. In this study, the time-convolution layer used a 1 × 128 kernel to capture temporal fluctuations and frequency-related information, followed by a depthwise convolution layer to model spatial information across channels, with a depth multiplier of D = 2. A pointwise convolution layer was then used to fuse the spatiotemporal features and improve model expressiveness. During training, the input signals were downsampled by a factor of 4 and Z-score standardized for each trial. A dropout rate of 0.25 was applied before the fully connected layer, and L2 regularization with a coefficient of 0.005 was introduced. The model was trained using the Adam optimizer with a learning rate of 0.001 for 50 epochs, and performance was evaluated using 5-fold cross-validation.

### 2.7. Primary Trial-Wise Cross-Validation and Model Evaluation

(1) Model training and evaluation strategy

For the primary within-dataset analysis, each trial was treated as an independent sample. Repeated five-fold cross-validation (10 repetitions) was used for model training and performance evaluation to reduce the influence of random data partitioning. Because trials from the same participant could be assigned to both the training and test folds, this procedure evaluated trial-level discrimination within the present dataset and was not interpreted as an estimate of subject-independent generalizability. Classification accuracy is reported as mean ± standard deviation. Within each cross-validation iteration, all supervised data-dependent steps, including feature standardization, PLV feature selection, FBCSP spatial-filter estimation, and classifier fitting, were performed using the training fold only. The corresponding parameters were then applied to the held-out test fold.

(2) Feature processing and leakage prevention

To ensure unbiased model evaluation, a strict nested feature-processing pipeline was implemented within the cross-validation framework for conventional ML models. Feature standardization and PLV feature reduction were both performed exclusively on the training folds, and the test folds were strictly isolated during processing. In the feature-selection stage, Elastic Net regularization (α = 0.5) was applied to handle highly correlated PLV features. The regularization parameter λ was dynamically optimized through internal 5-fold cross-validation and only features with non-zero coefficients were retained. EEGNet, as an end-to-end model, directly took the preprocessed time-series signals as input for automatic feature extraction. Although it did not involve the handcrafted feature-selection procedure described above, it followed the same data-partitioning scheme to ensure fair comparison.

This training-only principle was consistently applied in both the repeated five-fold cross-validation and the supplementary LOSO-CV procedure; in LOSO-CV, all preprocessing, feature extraction, and model training parameters were learned exclusively from the training participants, and the held-out participant’s data were not used in filter design, feature selection, or classifier training.

(3) Multi-dimensional decoding task analysis

The study included two levels of classification analysis. First, conditional discriminability analysis was performed to evaluate the classification performance of each model across the three shooting conditions. Second, shooting-performance discriminability analysis focused on decoding high- and low-performance trials within the same condition, systematically examining model performance across different frequency bands and time windows, as well as the end-to-end classification capability of EEGNet. For these two decoding tasks, the study further combined the Gini importance of the random forest model, the absolute coefficients of Elastic Net, and the spatial filters of FBCSP to examine feature contribution and importance from the perspectives of channels, connections, and spatial patterns, respectively.

In addition, statistical tests were conducted for band power and PLV features to provide biological significance support for the ML results. Detailed analyses are provided in the Appendix A.

### 2.8. Supplementary Subject-Independent Evaluation

As a supplementary subject-independent evaluation of the shooting-performance classification task, LOSO-CV was conducted using RF classifiers separately for band-power, PLV-based functional-connectivity, and FBCSP features. In each iteration, all trials from one participant were held out as the test set, while data from the remaining participants were used for model development. This procedure was repeated until each participant had served as the held-out test participant once.

All supervised data-dependent procedures, including feature standardization, PLV feature selection, FBCSP spatial-filter estimation, and classifier fitting, were performed using the training participants only within each LOSO-CV iteration. Data from the held-out participant were used exclusively for testing. LOSO-CV was used to evaluate generalization to previously unseen shooters and to quantify the influence of inter-individual variability; it was not expected to eliminate the distribution differences between participants.

### 2.9. Exploratory Decision-Score and Participant-Level Analyses Preocedures

Two additional exploratory analyses were conducted. First, because the binary model in Experiment 2 was trained only in high- and low-performance trials, the trained model was subsequently applied to medium-performance trials as an exploratory continuity check. Soft decision scores for the high-performance class were compared across the high-, medium-, and low-performance groups to examine their relative ordering along the learned binary decision function. This analysis was not intended to validate three-class or full-continuum classification.

Second, participant-level variability was examined descriptively within the trial-wise five-fold cross-validation framework. A five-fold partition stratified by participant identifier was generated to ensure that each participant contributed trials to every fold. In each fold, an RF classifier was trained in the training trials and used to predict the held-out test trials. The held-out predictions were then grouped by participant to calculate participant-specific accuracies. Ten RF training runs were performed using the same five-fold partition, and the accuracies were averaged across folds and runs. Because other trials from each participant were also present in the training folds, these results were interpreted as exploratory descriptive summaries rather than subject-independent validation performance.

## 3. Results

### 3.1. Conditional Discriminability

#### 3.1.1. Conditional Discriminability Based on Band Power Feature

Figure 2a shows the classification results of ML models based on band power features for EEG across different conditions. Figure 2(a-1) presents classification accuracy using fused frequency bands across different conditions. As shown in the figure, the RF model achieved relatively higher classification accuracy, with 74 ± 0.85% for normal vs. noise, 84 ± 0.49% for normal vs. low light, and 86 ± 0.56% for noise vs. low-light comparisons. Figure 2(a-2) shows the classification accuracy of different frequency-band features in the RF model. Across the three condition comparisons, the beta band consistently achieved the highest classification accuracy, followed by the gamma band. Figure 2(a-3) shows the channel-importance scalp topographies for beta-band features classified by the RF model across different conditions. For the normal vs. noise comparison, the mid-occipital and right temporoparietal regions contributed more strongly. For the normal vs. low-light comparison, the right frontal and frontotemporal regions contributed more strongly. For the noise vs. low-light comparison, the right prefrontal and frontal regions contributed more prominently.

#### 3.1.2. Conditional Discriminability Based on PLV Functional Connectivity Features

Figure 2b shows the classification results of ML models based on PLV functional connectivity features for EEG across different conditions. Figure 2(b-1) presents the classification accuracy using fused frequency bands across the different conditions. As shown in the figure, the SVM model achieved relatively higher classification accuracy, with 66 ± 1.25% for normal vs. noise, 83 ± 0.56% for normal vs. low light, and 82 ± 0.89% for noise vs. low-light comparisons. Figure 2(b-2) shows the classification accuracy of PLV-based features in the SVM model across different frequency bands. Across the three condition comparisons, the beta band consistently achieved the highest classification accuracy, followed by the gamma band. Figure 2(b-3) shows the connection importance after Elastic Net regularization-based feature selection for cross-condition classification. In the figure, line thickness represents feature importance, with red indicating a positive correlation with the former class and blue indicating a positive correlation with the latter class.

In the normal vs. noise comparison, the connections contributing significantly to the normal condition were Fp1-C3, P3-CP2, F8-C4, Fp2-P7, F7-FC5, and T8-CP6; the connections contributing significantly to the noise condition were Fp1-F4, FC1-FC2, F4-C4, CP2-PO4, and Fp1-CP2. In the normal vs. low-light comparison, the significant connections for the normal condition were O1-O2, CP6-P8, CP5-P7, FC5-C3, F2-FC2, CP1-CP2, and F7-P4; the significant connections for the low-light condition were FC1-FC2, Fp2-F8, Fz-Cz, F3-C3, PO4-O2, and C3-Cz. In the noise vs. low-light comparison, the significant connections for the noise condition were CP6-P8, CP5-P7, O1-O2, PO3-P8, FC5-C3, C3-CP1, and Fz-C4; the significant connections for the low-light condition were Fp1-F7, Fp2-F8, P3-PO3, PO4-O2, F8-FC6, and F4-FC1.

#### 3.1.3. Conditional Discriminability Based on FBCSP Features

Figure 2c shows the classification results of ML models based on FBCSP features for EEG across different conditions. Figure 2(c-1) presents the overall classification accuracy across different conditions. As shown in the figure, the SVM model achieved relatively higher classification accuracy, with 81 ± 0.1% for normal vs. noise, 91 ± 0.17% for normal vs. low light, and 90 ± 0.69% for noise vs. low-light comparisons. Figure 2(c-2) shows the classification accuracy of CSP features across different frequency bands in the SVM model. Across the three condition comparisons, the beta and gamma bands consistently achieved higher classification accuracy. Due to space limitations, the scalp topographies of spatial pattern weights for cross-condition classification are provided in Appendix A Figure A7.

### 3.2. Shooting Performance Classification Within the Same Condition

#### 3.2.1. Clustering and Contribution of Behavioral Metrics

Figure 3 illustrates the K-means clustering analysis and evaluation of shooting behavioral indices across different conditions. Figure 3a shows the spatial distribution of the clustering results based on t-SNE dimensionality reduction. In the figure, the horizontal and vertical axes represent two dimensions after dimensionality reduction in the behavioral feature space across different conditions; blue indicates the high-performance class, whereas red indicates the low-performance class.

During K-means clustering, the variance of each feature across all cluster centroids was recorded. A larger variance indicates a greater difference in that feature among cluster centroids, suggesting stronger discriminative capacity. The results are shown in Figure 3b. Overall, the contribution of each behavioral index to clustering varied. SCORE and COG played the most prominent roles, indicating that multiple behavioral indices jointly influenced the clustering outcome. Figure 3c presents the comparison of clustering silhouette coefficients across different conditions. The mean silhouette coefficients were 0.583 in the normal condition, 0.533 in the noise condition, and 0.630 in the low-light condition. These values indicate a moderate degree of separation among behavioral clusters. The subsequent binary EEG classification was restricted to the high- and low-performance clusters; therefore, the results reflect discriminability between behaviorally separated extreme groups. In the present analysis, these behavioral-feature contributions were used only to interpret the construction of the performance labels. Behavioral indices were not used as predictors in the EEG classification models.

#### 3.2.2. Discriminability of Shooting Performance Based on Power Features

Figure 4 shows the classification evaluation of shooting-performance EEG based on band power features across the three conditions. Figure 4a presents the confusion matrices and fused-band classification accuracy of different models across the three conditions. The RF model achieved better performance, with classification accuracies of 78 ± 0.73% in the normal condition, 74 ± 0.81% in the noise condition, and 63 ± 0.83% in the low-light condition. Figure 4b shows the ROC curves of different models across the three conditions, with the area under the curve (AUC) used to evaluate overall model performance. As shown in Figure 4a,b, the RF model performed best in the normal condition, with a recall of 68%, specificity of 91%, and AUC of 0.83, followed by the noise condition, with the lowest performance observed in the low-light condition. Figure 4c shows the classification accuracy of band power features across different frequency bands in the three conditions. Overall, the beta band achieved the highest accuracy, followed by the gamma band. Figure 4d shows the scalp topographies of feature importance for the RF classification model based on beta-band features. Darker red indicates higher feature importance during classification. The beta-band spatial discriminative patterns showed condition-specific shifts. In the normal condition, high-weight discriminative features showed an asymmetric distribution, with the main contribution concentrated in the left prefrontal region and a secondary contribution in the right parieto-occipital region. In the noise condition, the discriminative pattern shifted toward the midline and the contralateral side, mainly involving the mid-frontal region, left occipital region, and parieto-occipital junction. In the low-light condition, the discriminative features showed a more distributed pattern, with the most prominent high-weight region located in the right frontal pole and additional weights observed in the central and left parieto-occipital regions.

#### 3.2.3. Discriminability of Shooting Performance Based on PLV Functional Connectivity Features

Figure 5 shows the classification evaluation of shooting-performance EEG based on PLV-based functional connectivity features across the three conditions. Figure 5a presents the confusion matrices and fused-band classification accuracy of different models across the three conditions. The SVM and RF models achieved comparable performance, with classification accuracies of 78 ± 0.61% in the normal condition, 75 ± 0.88% in the noise condition, and 63 ± 1.1% in the low-light condition. Figure 5b shows the ROC curves of different models across the three conditions, with the area under the curve (AUC) used to evaluate overall model performance. As shown in Figure 5a,b, the SVM model performed best in the normal condition, with a recall of 67%, specificity of 94%, and AUC of 0.83, followed by the noise condition, with the lowest performance observed in the low-light condition. Figure 5c shows the classification accuracy of PLV-based features across different frequency bands in the three conditions. Overall, the beta band achieved the highest accuracy, followed by the gamma band. Figure 5d shows the spatial distribution of important brain network connections identified by Elastic Net regularization-based feature selection, and Figure 5e shows the top 15 most important connections. In the normal condition, the most important connections included C3-FC5, CP2-F3, O2-T8, Cz-Fz, and T7-F3. In the noise condition, the most important connections included FC6-F4, P8-T8, FC1-F3, P7-T7, Pz-F4, and CP6-O1. In the low-light condition, the most important connections included F4-F3, CP6-O2, O1-P3, P4-CP2, and O1-Oz.

#### 3.2.4. Discriminability of Shooting Performance Based on FBCSP Features

Figure 6 shows the classification evaluation of shooting-performance EEG based on FBCSP features across the three conditions. Figure 6a presents the confusion matrices and fused-band classification accuracy of different ML models across the three conditions. The SVM model achieved the best performance, with fused-band classification accuracies of 81 ± 0.69% in the normal condition, 75 ± 0.77% in the noise condition, and 63 ± 1.3% in the low-light condition. Figure 6b shows the ROC curves of different ML models across the three conditions, with AUC used to evaluate overall model performance. As shown in Figure 6a,b, the SVM model performed best in the normal condition, with a recall of 67%, specificity of 91%, and AUC of 0.86, followed by the noise condition, with the lowest performance observed in the low-light condition. Figure 6c shows the classification accuracy of FBCSP features across different frequency bands and time windows in the three conditions. Overall, the beta band achieved the highest classification accuracy, followed by the gamma band. In terms of time windows, higher classification accuracy was observed in the middle and late time windows. The FBCSP method divided the signals into nine frequency bands, and the classification accuracy across different time windows for these nine bands is provided in the Appendix A. Figure 6d shows the scalp topographies of spatial pattern weights for the two classes obtained from FBCSP in the normal condition. A1 indicates brain regions with high discriminability for the high-performance class, and A2 indicates brain regions with high discriminability for the low-performance class. The A1 spatial pattern was concentrated in the left frontal cortex and left central region. The A2 spatial pattern was concentrated in the left posterior parietal region and the left centro-parietal junction. The scalp topographies of spatial pattern weights for the noise and low-light conditions are provided in Appendix A Figure A8.

### 3.3. Supplementary Subject-Independent Classification Performance

Figure 7 presents LOSO-CV accuracies for shooting-performance classification across the three conditions. In contrast to trial-wise five-fold cross-validation, LOSO-CV evaluated performance when the test participant was not represented in the training data. Using band-power features with the RF model, accuracies were 59.4%, 61.0%, and 70.1% under normal, noise, and low-light conditions, respectively. Using PLV functional connectivity features with the RF model, accuracies were 61.7%, 60.9%, and 59.8%, respectively. Using FBCSP features with the RF model, accuracies were 61.6%, 61.1%, and 59.8%, respectively. Overall, LOSO-CV performance was moderate and lower than the corresponding trial-wise results, indicating modest cross-subject discriminability and substantial inter-individual variability.

### 3.4. Exploratory Decision-Score and Participant-Level Analyses

Figure 8 shows the distribution of RF model prediction scores and participant-level classification accuracies across the three conditions. Figure 8a shows the predicted scores for the high-performance class after EEG features from the three performance levels (high, medium, and low), generated by behavioral-index clustering, were input into the trained RF model. These scores were examined exploratorily to assess continuity along the learned binary decision function across the three behaviorally defined performance groups; they were not treated as independent validation of three-class or full-continuum classification. For FBCSP features, the predicted scores for the high-, medium-, and low-performance trials were 0.79, 0.30, and 0.16 in the normal condition; 0.75, 0.25, and 0.15 in the noise condition; and 0.77, 0.45, and 0.30 in the low-light condition, respectively. For PLV functional connectivity features, the corresponding scores were 0.71, 0.31, and 0.20 in the normal condition; 0.71, 0.31, and 0.20 in the noise condition; and 0.77, 0.50, and 0.31 in the low-light condition, respectively.

Figure 8b shows the distribution of mean participant-level classification accuracies and kernel density curves across the three conditions using five-fold cross-validation. In the normal condition, 15 participants achieved classification accuracies above 0.80 for both FBCSP and PLV functional connectivity features. In the noise condition, 14 participants achieved accuracies above 0.80 for both feature types. In the low-light condition, 17 participants achieved accuracies above 0.60 for FBCSP features, whereas 21 participants achieved accuracies above 0.60 for PLV functional connectivity features.

## 4. Discussion

This study examined the discriminability of pre-shot EEG signals under normal, noise, and low-light conditions, as well as EEG patterns associated with different shooting-performance levels within each condition. The results showed that band-power, PLV-based functional-connectivity, and FBCSP features all contained information relevant to the two classification tasks. FBCSP showed the strongest overall within-dataset performance. Beta-band features showed relatively consistent discriminative contributions across tasks, whereas gamma-band features contributed to several analyses but required cautious interpretation. However, the lower LOSO-CV performance indicated that cross-subject generalization remained limited.

### 4.1. Discriminability of Different Shooting Conditions Across Feature Types

The present results show that models based on band power, PLV functional connectivity, and FBCSP features were all able to distinguish the normal, noise, and low-light conditions to some extent. Among the three feature types, FBCSP achieved the best classification performance, suggesting that spatial pattern information plays an important role in identifying EEG differences across shooting conditions. In contrast, band power and PLV functional connectivity features also showed certain discriminative ability, but their performance was more strongly influenced by feature dimensionality, noise sensitivity, and model type.

At the model level, FBCSP and PLV features performed better with the SVM model, whereas band power features performed relatively better with the RF model. This suggests that the statistical properties and structural forms of the features may affect classifier compatibility. Band power features have relatively low dimensionality but may be more directly affected by conditional interference and non-task-related fluctuations; RF, through ensemble learning and random feature selection, may be better suited to handling such noisy features [41]. By contrast, FBCSP and PLV features contain stronger spatial or connectivity structure, and SVM may have an advantage when dealing with these high-dimensional discriminative features [42].

At the frequency-band level, beta-band and gamma-band features showed relatively high discriminative contributions across the classification tasks. The relatively consistent contribution of beta-band features may have a neurophysiological basis. Beta activity over sensorimotor and frontoparietal regions has been associated with maintenance of the current motor state, motor preparation, and regulation of movement execution [43]. During the pre-shot period, shooters must maintain postural and aiming stability while preparing a highly controlled trigger action. Beta-band modulation may therefore reflect differences in sensorimotor stabilization, motor-set maintenance, and visuomotor integration under different environmental conditions and performance levels. In addition, beta activity has been linked to top-down attentional control and suppression of task-irrelevant processing, which may be particularly relevant when shooters must maintain stable attention under noise or reduced visual information. Nevertheless, the present feature-importance results identify discriminative information for classification and should not be interpreted as demonstrating a causal role of beta activity in shooting performance.

Gamma-band features also contributed to classification in several analyses. At a neurophysiological level, gamma activity has been associated with local cortical processing, attentional selection, sensory integration, and motor-related information processing [43]. In the present task, gamma-band discriminability may therefore be related to differences in visuomotor integration, attentional allocation, or motor preparation during the pre-shot interval. However, this interpretation requires particular caution. Standing pistol shooting involves sustained postural control and subtle facial, jaw, neck, and trigger-related muscle activity, and the present study did not include dedicated EMG recordings. Thus, residual muscle activity cannot be fully excluded from signals in the gamma band. Gamma-band findings should consequently be interpreted as discriminative signal features associated with shooting preparation, rather than as unequivocal evidence of cortical gamma mechanisms.

From the perspective of spatial distribution and connectivity patterns, the channels and connections contributing most strongly differed across condition comparisons. Normal vs. noise, normal vs. low light, and noise vs. low-light comparisons involved different combinations of occipital, frontal, frontotemporal, and sensorimotor-related regions, suggesting that noise and low-light conditions may correspond to different neural modulation patterns. Under noise conditions, the model captured more features related to auditory interference suppression, visual attention maintenance, and sensorimotor integration [44]. Under low-light conditions, the increased importance of frontal and frontotemporal regions may reflect additional attentional control and motor preparation demands when visual information is restricted. These results are broadly consistent with previous studies on how conditional interference affects brain functional networks during shooting preparation, but they should still be interpreted as differences in discriminative features rather than direct mechanistic evidence.

### 4.2. Discriminability of Shooting Performance Within the Same Condition

For shooting-performance classification, K-means clustering of multidimensional behavioral indices was used to construct high-, medium-, and low-performance labels. The high- and low-performance trials showed a degree of separation in behavioral space, supporting their use in the subsequent binary analysis. However, these labels were operational categories constructed from behavioral features rather than naturally defined physiological classes. The classification results should therefore be interpreted as the discriminability of EEG patterns associated with behaviorally distinct performance groups.

The seven behavioral indices were used to construct the high-, medium-, and low-performance labels through K-means clustering, whereas the behavioral-contribution analysis quantified the relative influence of each index on the clustering results. These behavioral indices and their contribution values were not included as predictors in the EEG classifiers or used for EEG feature selection in the present analysis. This distinction is important because directly using the variables that contributed to the construction of the performance labels as predictors in the same classification task could introduce circularity and information leakage.

The behavioral-contribution analysis nevertheless provides a potential basis for behavior-informed EEG feature selection and model training. In future studies, continuous behavioral dimensions such as shooting score, aiming stability, and trigger-control efficiency could be used to identify EEG frequency bands, channels, functional connections, or spatial patterns that are consistently associated with specific performance components. These behavioral measures could also serve as continuous regression targets or auxiliary tasks in a multi-task learning framework. Behavior-informed regularization or multimodal models may further improve the interpretability of EEG decoding by linking discriminative EEG features to specific aspects of shooting performance. These approaches were not implemented in the present study and should be evaluated using strictly separated training and test procedures.

All three EEG feature types distinguished high- and low-performance trials within the same condition to some extent. FBCSP showed the strongest overall performance, indicating that class-related spatial patterns were particularly informative for this task. Band-power and PLV features also showed discriminative performance, especially in the beta band and gamma band. The possible neurophysiological relevance of these frequency ranges was discussed above; however, their contributions should primarily be interpreted as information used by the classifiers rather than as evidence of causal neural mechanisms.

Although the detailed EEGNet results are reported in Appendix A.5, its lower performance relative to the handcrafted-feature models merits consideration. EEGNet was included as a lightweight end-to-end benchmark rather than as a fully optimized deep-learning model. It was trained using fixed architecture and fixed training parameters, without extensive architecture comparison or hyperparameter search. Its lower performance should therefore be interpreted in the context of the present dataset and model configuration. The limited number of participants and substantial inter-individual EEG variability may have constrained EEGNet’s ability to learn stable and participant-invariant spatiotemporal representations directly from the EEG time series.

By contrast, FBCSP performs frequency-band decomposition and supervises spatial filtering before classification. It emphasizes spatial patterns that maximize between-class variance differences within each frequency band, thereby reducing the burden on the classifier to learn spectral and spatial structure directly from the raw signals. This prior feature construction may be advantageous for the present dataset and may explain the stronger discriminative performance of FBCSP under the current model configuration. However, these findings do not imply that FBCSP is universally superior to EEGNet or that conventional machine learning methods are generally better than deep-learning methods for small-sample EEG classification.

Across conditions, performance classification was highest under the normal condition, decreased under noise, and was lowest under low-light conditions. EEG differences between high- and low-performance trials may become less pronounced when visual information is restricted. Under low-light conditions, shooters may recruit additional attentional-maintenance, visual-compensation, and motor-preparation resources, thereby reducing differences between performance levels. The feature-importance and connectivity results were consistent with condition-dependent patterns: normal-condition discrimination involved frontal, central, and parieto-occipital features, whereas noise and low-light conditions showed shifts in frontal, parieto-occipital, and sensorimotor-related contributions. These results suggest that performance-related EEG discriminability may depend on the environmental demands of the shooting task.

When medium-performance trials were re-entered into the trained binary model, their decision scores generally fell between those of the high- and low-performance trials. This exploratory result is consistent with continuity in the learned decision function; however, it does not remove the limitation that the primary binary models were trained and evaluated using extreme performance groups. The participant-level analysis also revealed substantial inter-individual variability.

Graph-theoretical measures showed statistically significant group-level differences at selected frequency bands and sparsity levels, but their single-trial classification accuracies were generally close to chance (Table A1 and Table A2 in Appendix A.2). Thus, group-level statistical differences did not necessarily provide stable features for single-trial decoding. Global graph measures may compress spatially specific and connection-specific information into a small number of summary statistics and may also be sensitive to network-construction choices. Detailed results, limitations, and potential improvements for graph-theoretical classification are provided in Appendix A.2.

Finally, the trial-wise five-fold accuracies should be interpreted as estimates of within-dataset trial-level discrimination rather than subject-independent performance because trials from the same participant could occur in both the training and test folds. In the supplementary LOSO-CV analysis of shooting-performance classification, accuracy ranged from 59.4% to 70.1% across feature types and shooting conditions. The reduction relative to trial-wise cross-validation indicates that participant-specific EEG characteristics and inter-individual variability remain substantial barriers to generalization to previously unseen shooters. Thus, LOSO-CV quantified the cross-subject generalization challenge but did not eliminate it.

### 4.3. Limitations and Future Directions

Several limitations should be acknowledged. First, the sample size and data source were limited. The data were collected using a single experimental platform under three laboratory conditions, and the primary trial-wise cross-validation analysis may have been influenced by participant-specific EEG characteristics. Although LOSO-CV was additionally used as a subject-independent baseline for the shooting-performance classification task, its lower accuracy indicated that inter-individual variability remains a major challenge. Future studies should validate the present framework in larger independent cohorts and investigate domain-adaptation and transfer-learning strategies for cross-subject EEG decoding. Candidate approaches include feature-space alignment methods, such as Euclidean or Riemannian alignment; distribution-discrepancy minimization or adversarial domain-adaptation methods; and few-shot calibration or fine-tuning using a small number of labeled trials from a new shooter. Zero-calibration approaches should be evaluated using strictly subject-independent data partitions without access to target-participant labels. For few-shot adaptation, calibration trials from the new participant should be kept separate from the trials used for final evaluation. These approaches should be compared directly with the LOSO-CV baseline reported in the present study.

Second, the performance-classification analysis retained only the high- and low-performance clusters and excluded the medium-performance cluster. This extreme-group design facilitated the examination of EEG differences between behaviorally distinct groups but likely increased class separability. Therefore, the binary results should not be interpreted as classification across the full continuum of shooting performance or as a three-class classification result. Future studies should retain intermediate-performance trials and evaluate multiclass, ordinal, regression-based, or behavior-informed multi-task models.

In addition, future studies should investigate behavior-informed EEG decoding using continuous and component-specific behavioral outcomes, while ensuring strict separation between label construction, behavioral-feature analysis, EEG feature selection, model training, and evaluation within each training fold.

Third, dedicated EOG and facial or neck EMG channels were not recorded. Although ICA-based correction was used to reduce ocular artifacts, residual muscle activity cannot be fully excluded from gamma-band features, and the use of Fp1/Fp2 as virtual EOG references may have affected genuine frontal EEG activity. Accordingly, gamma-band and frontal-channel findings should be interpreted as discriminative signal features rather than unequivocal neural effects. Future studies should acquire dedicated EOG and facial/neck EMG recordings and evaluate the robustness of these findings under alternative artifact-correction procedures [45].

Finally, the present study was based on offline laboratory data [46] and focused mainly on discriminative associations rather than causal mechanisms. The detailed graph-theoretical analyses also showed that group-level statistical differences did not necessarily translate into reliable single-trial classification, highlighting the sensitivity of such measures to network-construction choices and feature representation. Future work should evaluate graph-theoretical, spatial, and connectivity features under alternative network-construction procedures and assess their performance in real-time and subject-independent settings. Longitudinal designs and causal connectivity analyses may further clarify the mechanistic relevance of the identified EEG features. More broadly, these practical and methodological constraints may partly explain why ML applications remain relatively limited in shooting-related EEG research. Addressing these challenges will require larger independent or shared datasets, standardized experimental protocols and performance-label definitions, and transparent subject-independent validation procedures.

## 5. Conclusions

This study developed an ML classification framework for EEG signals recorded during shooting preparation and separately evaluated band-power, PLV-based functional-connectivity, and FBCSP features for distinguishing shooting conditions and shooting-performance levels within the same condition. The results showed that EEG patterns associated with different shooting conditions and performance levels were discriminable to some extent, with FBCSP combined with SVM achieving the best overall performance under the present experimental setting. Beta-band features showed relatively consistent discriminative contributions across the two classification tasks, while gamma-band features also contributed to several analyses but should be interpreted cautiously because residual muscle activity could not be fully excluded.

The trial-wise five-fold cross-validation results primarily demonstrate within-dataset trial-level discriminability rather than generalization to unseen shooters. In the shooting-performance classification task, the lower LOSO-CV accuracy relative to trial-wise cross-validation indicates that inter-individual variability remains an important limitation to subject-independent generalization. Overall, this study provides preliminary evidence of EEG discriminability for shooting environments and behaviorally defined performance groups, rather than validation of a deployable monitoring method. Further validation using larger independent cohorts, strict subject-independent evaluation, and appropriate subject-adaptation strategies is required before practical monitoring applications can be considered.

## Figures and Tables

**Figure 1:**
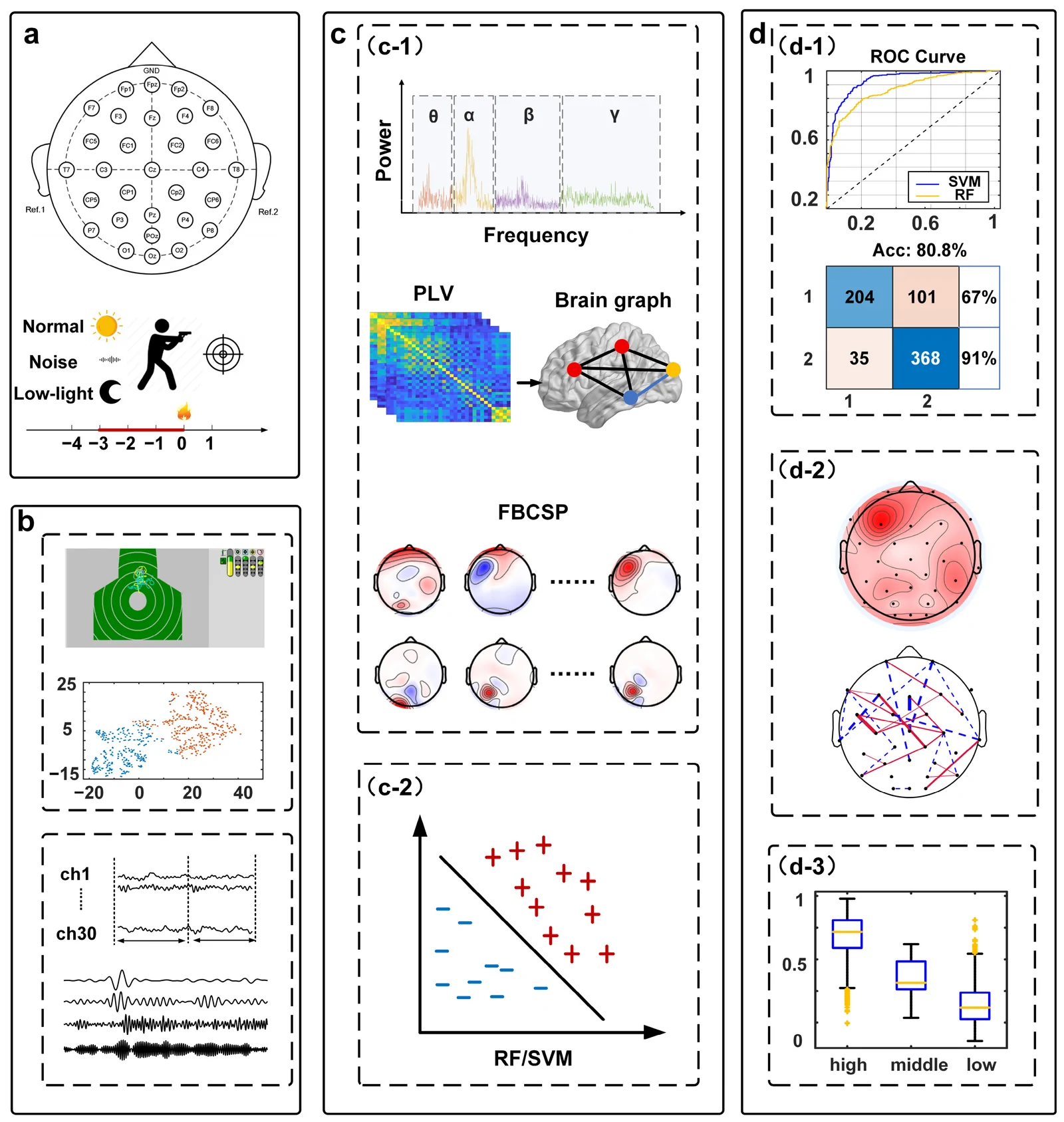
Data analysis workflow: (**a**) experimental data acquisition; (**b**) data preprocessing; (**c**) feature extraction and model training, including (**c-1**) extraction of band power, PLV-based functional connectivity, and FBCSP features, and (**c-2**) classification model training; (**d**) model performance analysis, including (**d-1**) ROC curve and confusion matrix analysis, (**d-2**) channel- and connection-importance analysis, (**d-3**) subject-independent LOSO-CV evaluation and additional analyses.

**Figure 2:**
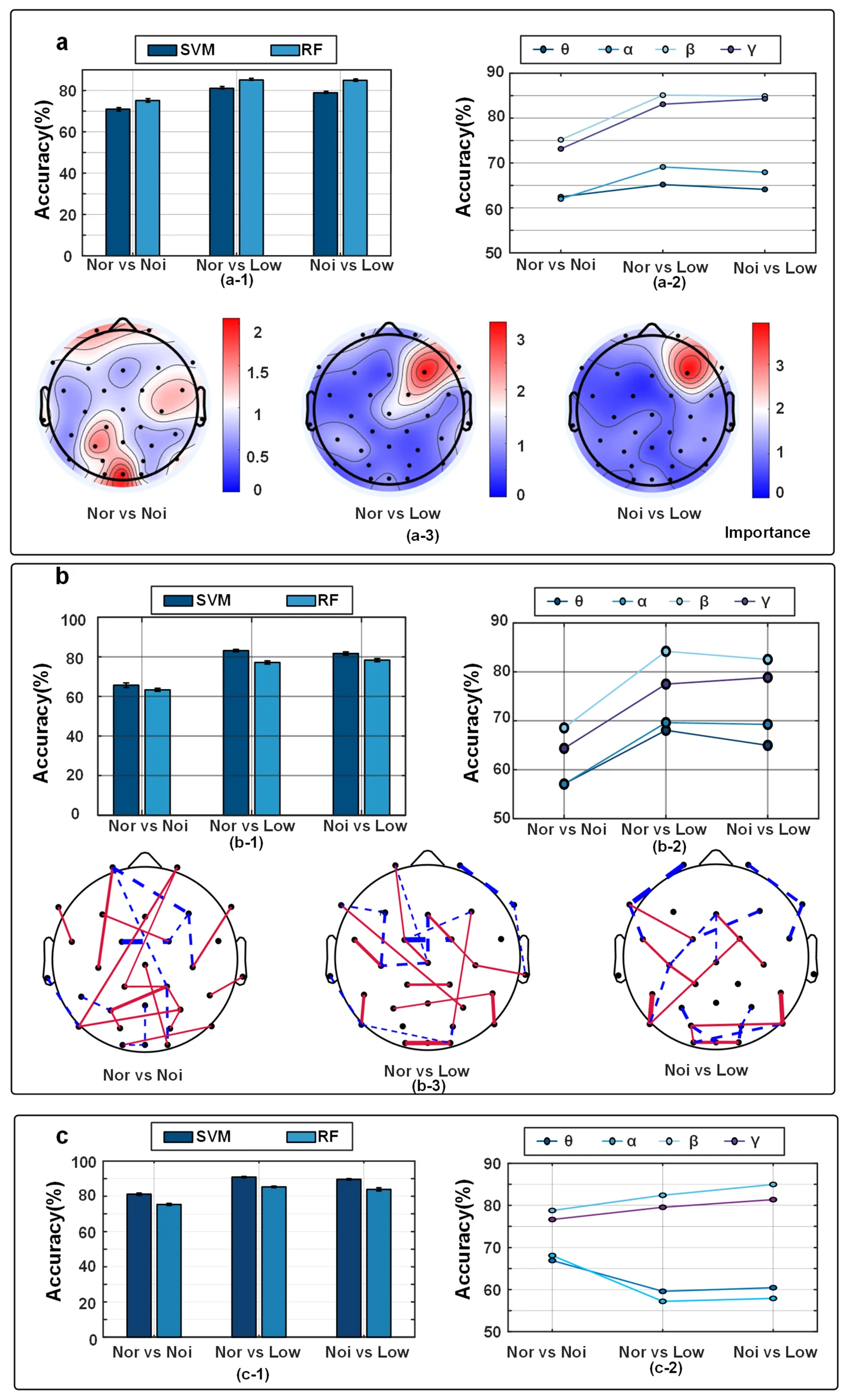
Classification results of EEG signals across the three conditions using ML models in different feature spaces. (**a**) Classification performance based on band power features: (**a-1**) fused-band classification accuracy across conditions; (**a-2**) comparison of RF model accuracy using single-band features; (**a-3**) scalp topographies of channel feature importance for beta-band RF classification. (**b**) Classification performance based on PLV features: (**b-1**) fused-band classification accuracy across conditions; (**b-2**) comparison of SVM model accuracy using PLV features across different frequency bands; (**b-3**) distribution of brain network connection importance based on Elastic Net feature selection (thicker lines indicate greater importance; red lines indicate positive correlation, corresponding to condition 1; blue lines indicate negative correlation, corresponding to condition 2). (**c**) Classification performance based on FBCSP features: (**c-1**) fused-band classification accuracy across conditions; (**c-2**) comparison of SVM model accuracy using CSP features across different frequency bands (Nor = normal, Noi = noise, Low = low light).

**Figure 3:**
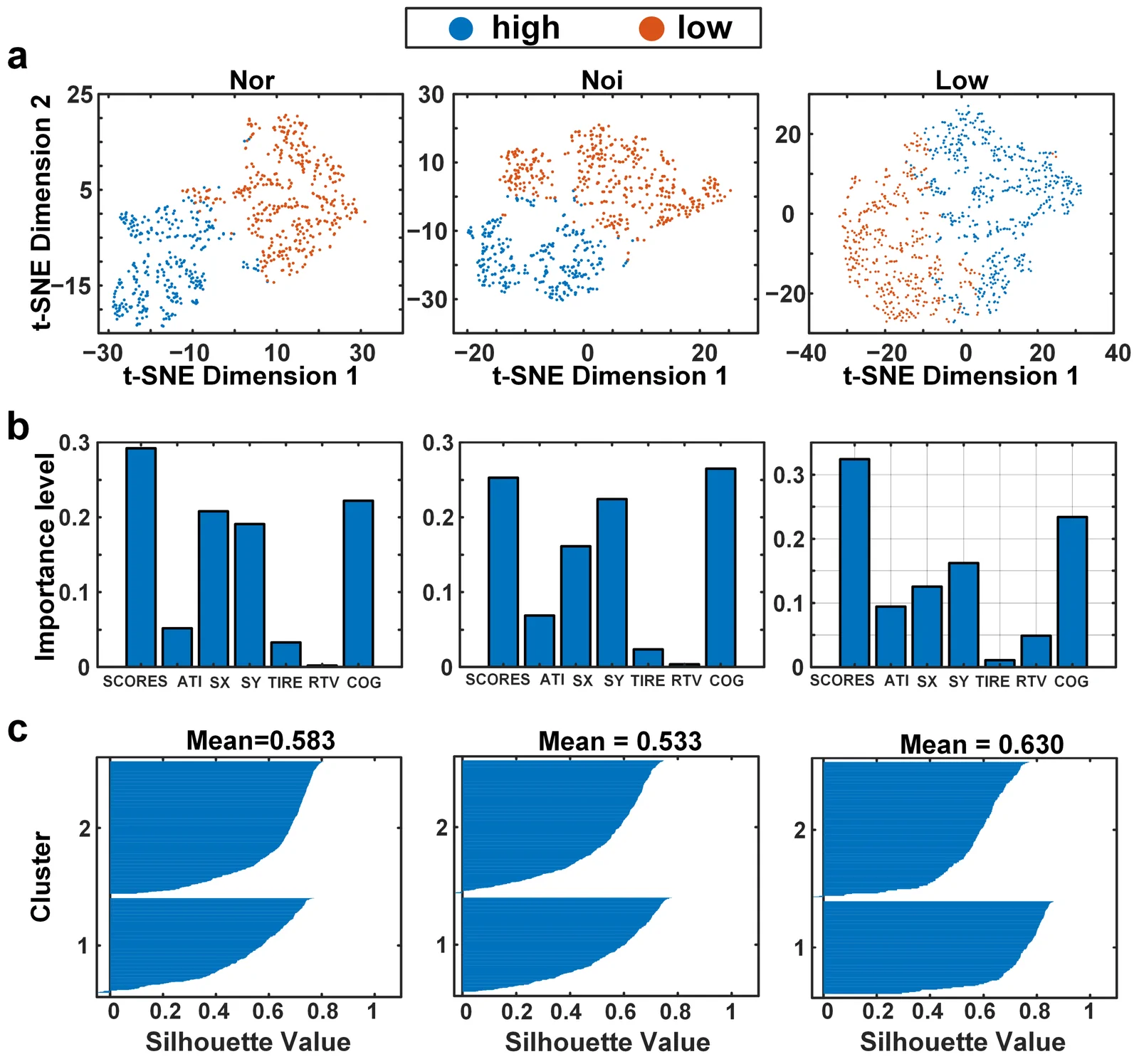
K-means clustering analysis and evaluation of shooting behavioral indices across the three conditions. (**a**) Spatial distribution of clustering results based on t-SNE dimensionality reduction. (**b**) Feature-importance evaluation of behavioral indices based on variance contribution. (**c**) Comparison of clustering silhouette coefficients across the three conditions (Nor = normal, Noi = noise, Low = low light).

**Figure 4:**
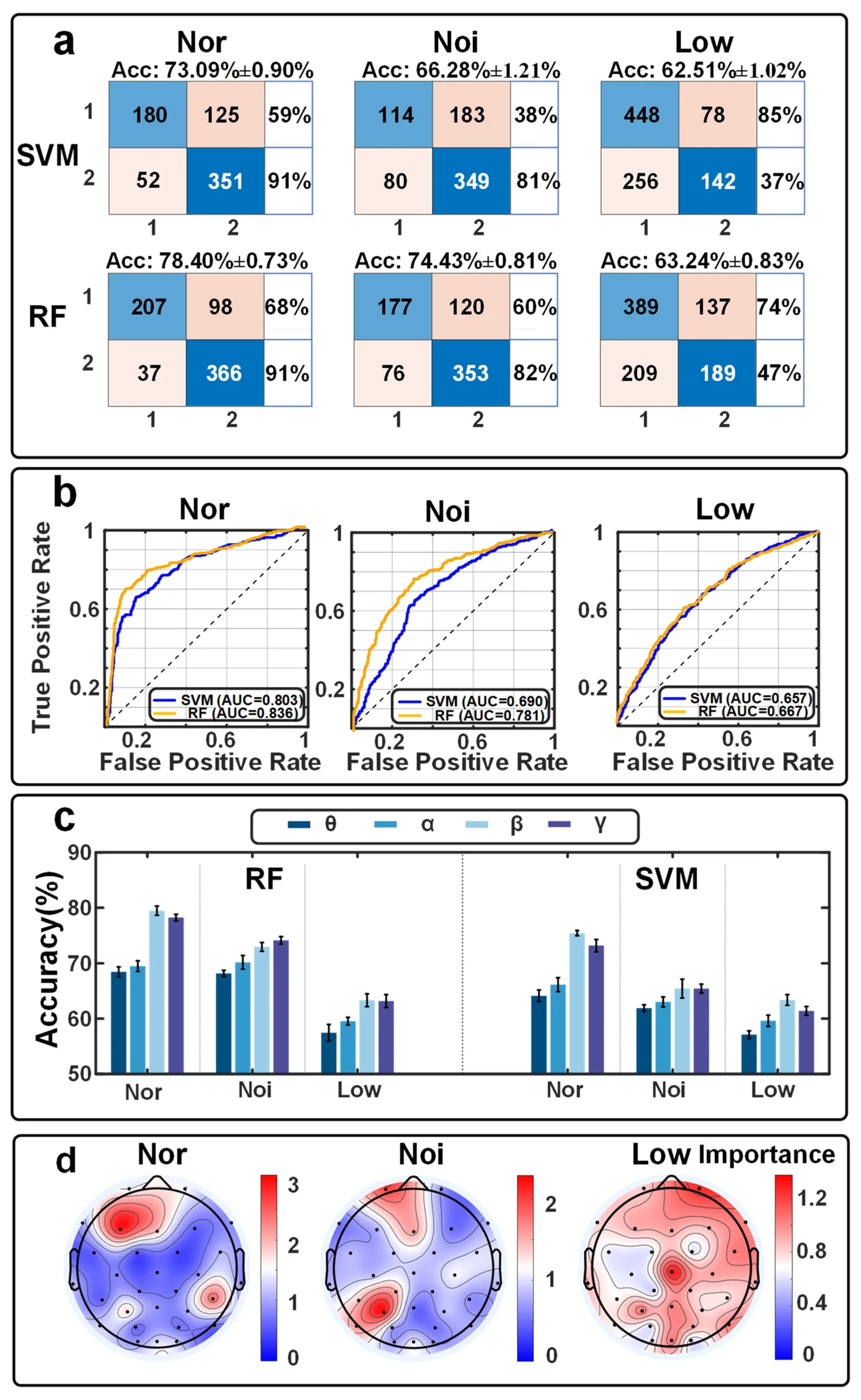
Classification evaluation of shooting-performance EEG based on band power features using ML models across the three conditions. (**a**) Confusion matrices of the two models under each condition. (**b**) ROC curves of the two models under each condition. (**c**) Comparison of classification accuracy across different frequency-band features under each condition. (**d**) Scalp topography of feature importance for the RF model based on beta-band features (Nor = normal, Noi = noise, Low = low light).

**Figure 5:**
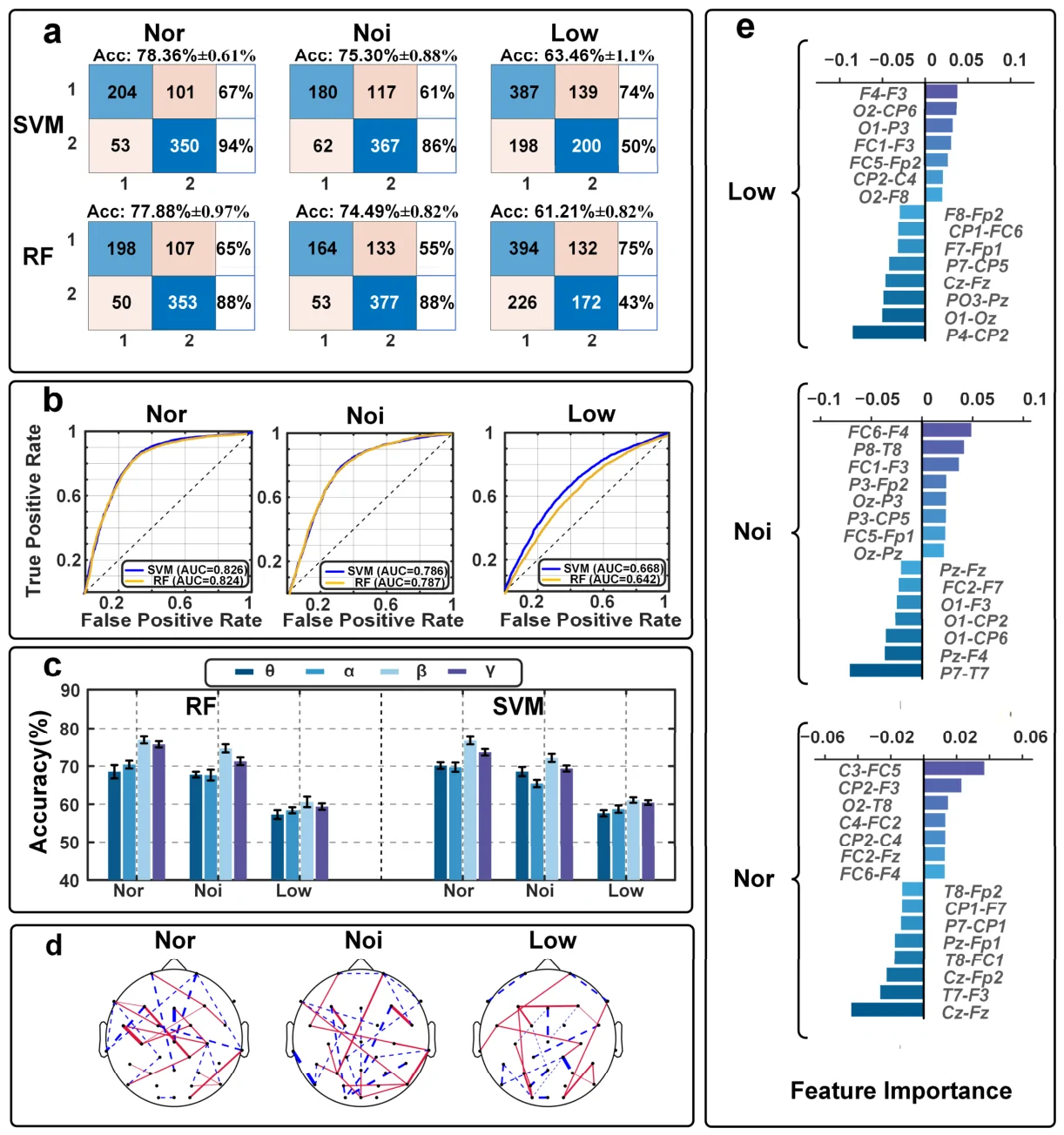
Classification evaluation of shooting-performance EEG based on PLV functional connectivity features using ML models across the three conditions. (**a**) Confusion matrices of the two models under each condition. (**b**) ROC curves of the two models under each condition. (**c**) Comparison of classification accuracy across different frequency-band PLV features under each condition. (**d**) Scalp topographies of important brain network connections under each condition based on Elastic Net regularization-based feature selection (red and blue lines indicate connections that are more prominent in the high- and low-performance classes, respectively; line thickness reflects connection importance). (**e**) Bar plots of the top 15 most important functional connections under each condition based on Elastic Net regularization-based feature selection (Nor = normal, Noi = noise, Low = low light).

**Figure 6:**
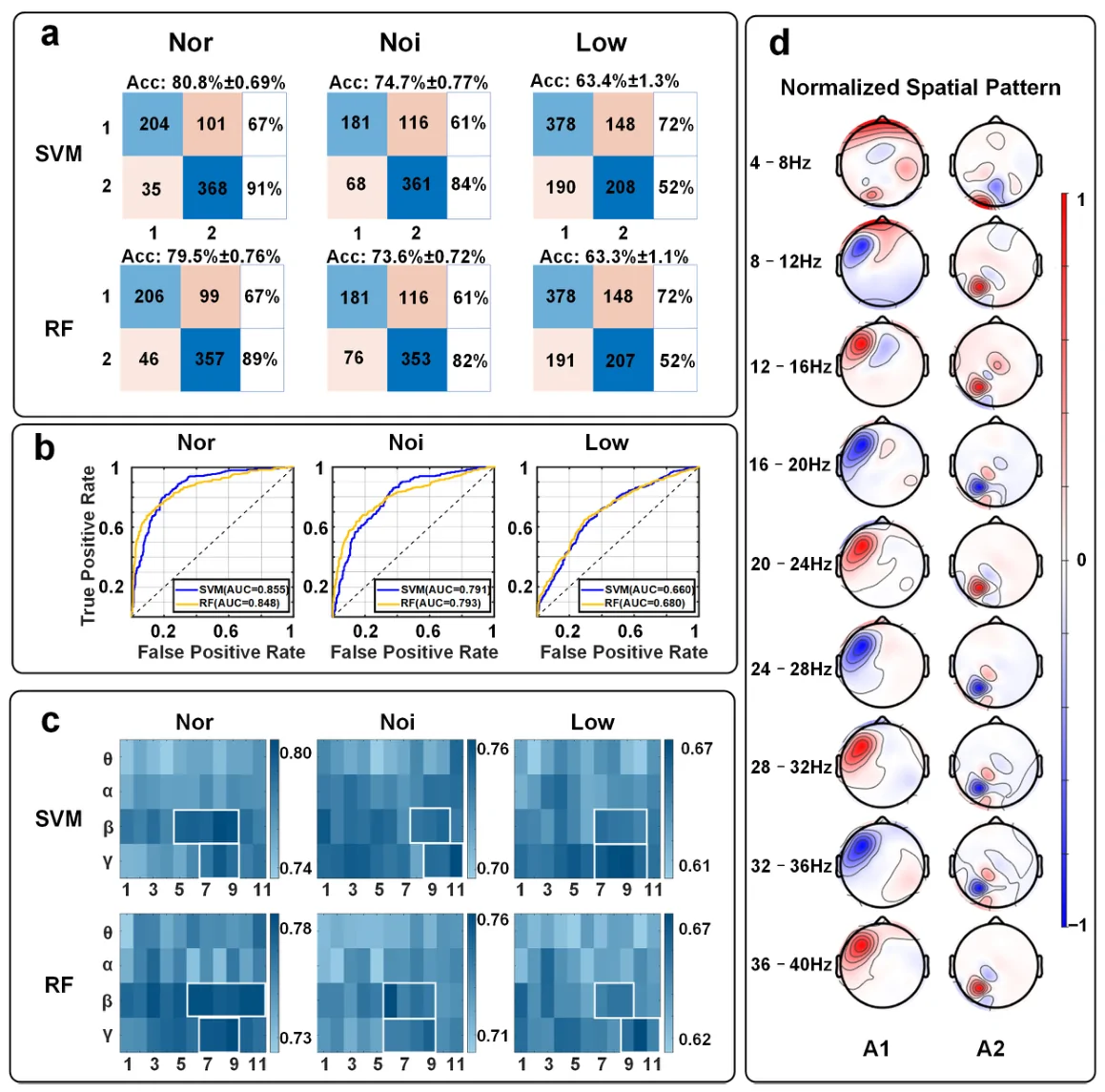
Classification evaluation of shooting-performance EEG based on FBCSP features using ML models across the three conditions. (**a**) Confusion matrices of the two models under each condition. (**b**) ROC curves of the two models under each condition. (**c**) Comparison of classification accuracy of CSP features across different frequency bands and time windows under each condition. (**d**) Scalp topographies of the two spatial patterns obtained from FBCSP in the normal condition (red and blue regions indicate increased and decreased feature weights, respectively) (Nor = normal, Noi = noise, Low = low light).

**Figure 7:**
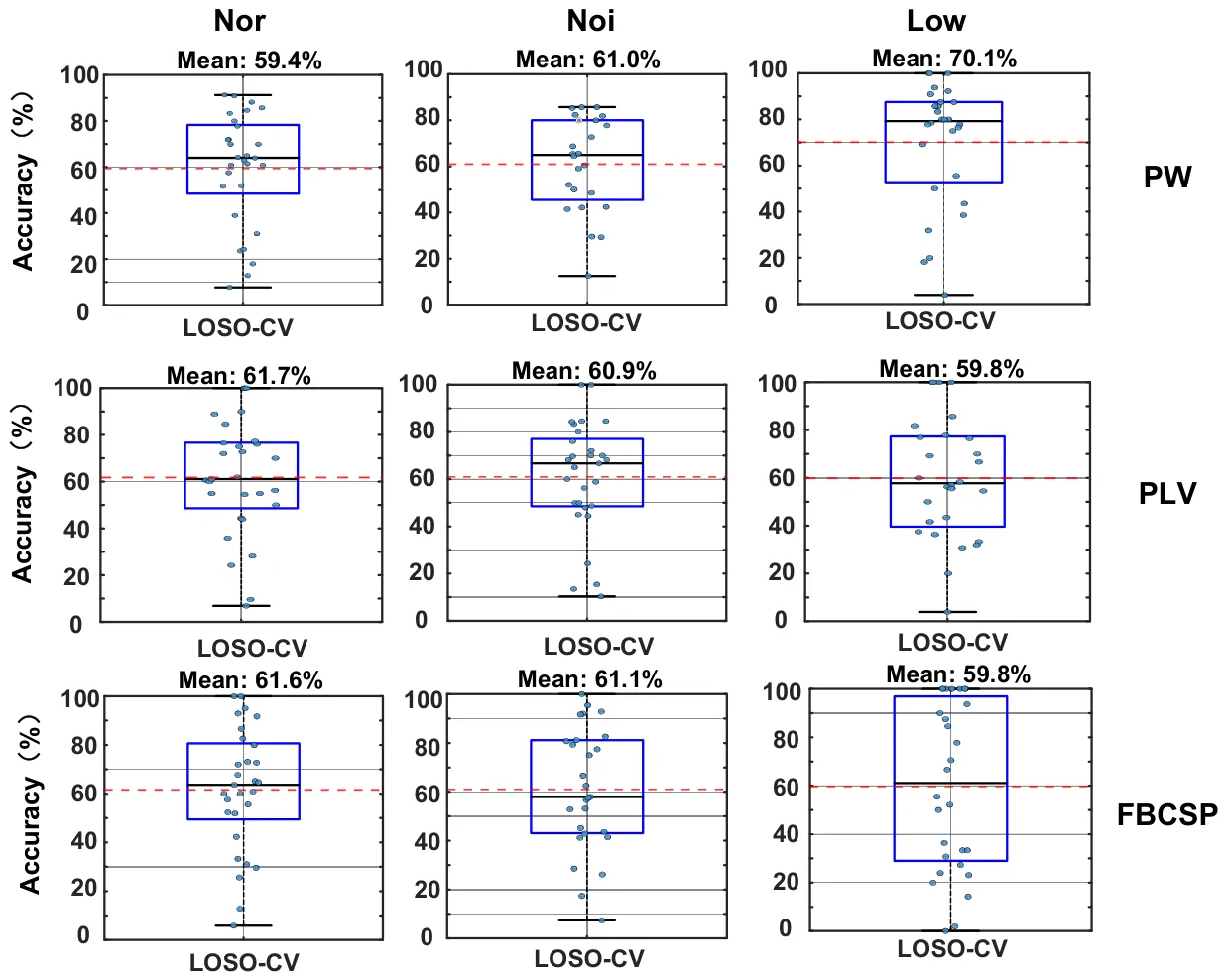
Participant-level shooting-performance classification accuracies obtained using supplementary LOSO-CV for the three EEG feature sets under normal, noise, and low-light conditions. Dots represent held-out participant accuracies; boxes indicate interquartile ranges; black horizontal lines indicate medians; and red dashed lines indicate means. (PW = frequency-band power, PLV = phase-locking value, FBCSP = filter bank common spatial pattern; Nor = normal, Noi = noise, Low = low light).

**Figure 8:**
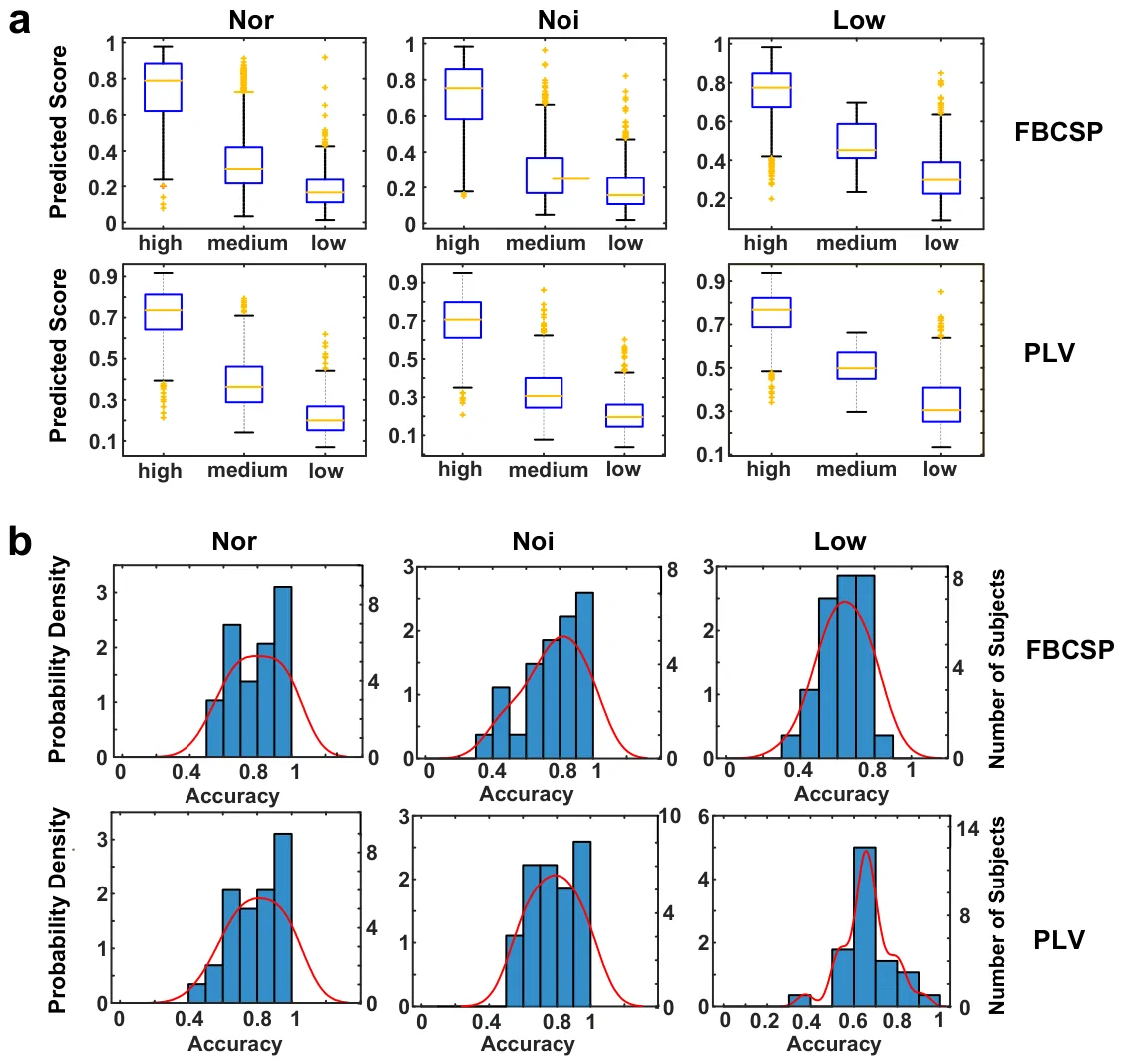
Distribution of RF model prediction scores and participant-level classification accuracies across the three conditions. (**a**) Comparison of prediction scores for high-, medium-, and low-performance levels across the three conditions. (**b**) Histogram and kernel density estimation curves of the mean classification accuracy across participants under the three conditions (Nor = normal, Noi = noise, Low = low light).

**Table 1:** Specific meanings of various shooting behavior indicators.

Indicator Parameter		Specific Meaning
SCORE		Shooting ring count (ranging from 5 to 10 rings; 0 rings if the target is not hit).
Gun-holding stability	SX	Average deviation of the horizontal movement of the aiming point; the smaller the value, the better.
	SY	Average deviation of the vertical movement of the aiming point; the smaller the value, the better.
Aiming ability	COG	Average center of gravity of the aiming curve; the closer to the bullseye, the better.
	ATI	Aiming time of the aiming point on the target.
Trigger pull control ability	RTV	Indicates whether the trigger is pulled smoothly. The smaller the value, the smaller the vibration of the aiming point when pulling the trigger.
	TIRE	Represents the quality, timing, and responsiveness of trigger pulling. A value of 3 is the best, and a value of 1 is the worst.

**Figure A1:**
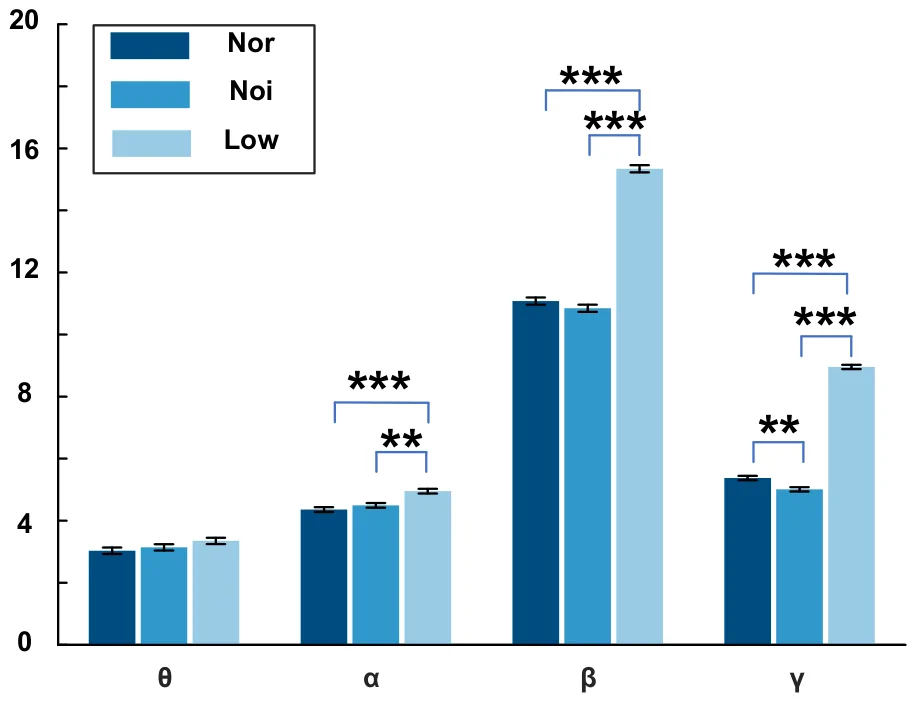
Inter-subject statistical differences in band power features across frequency bands under the three conditions (Nor = normal, Noi = noise, Low = low light). ** *p* < 0.01, *** *p* < 0.001.

**Figure A2:**
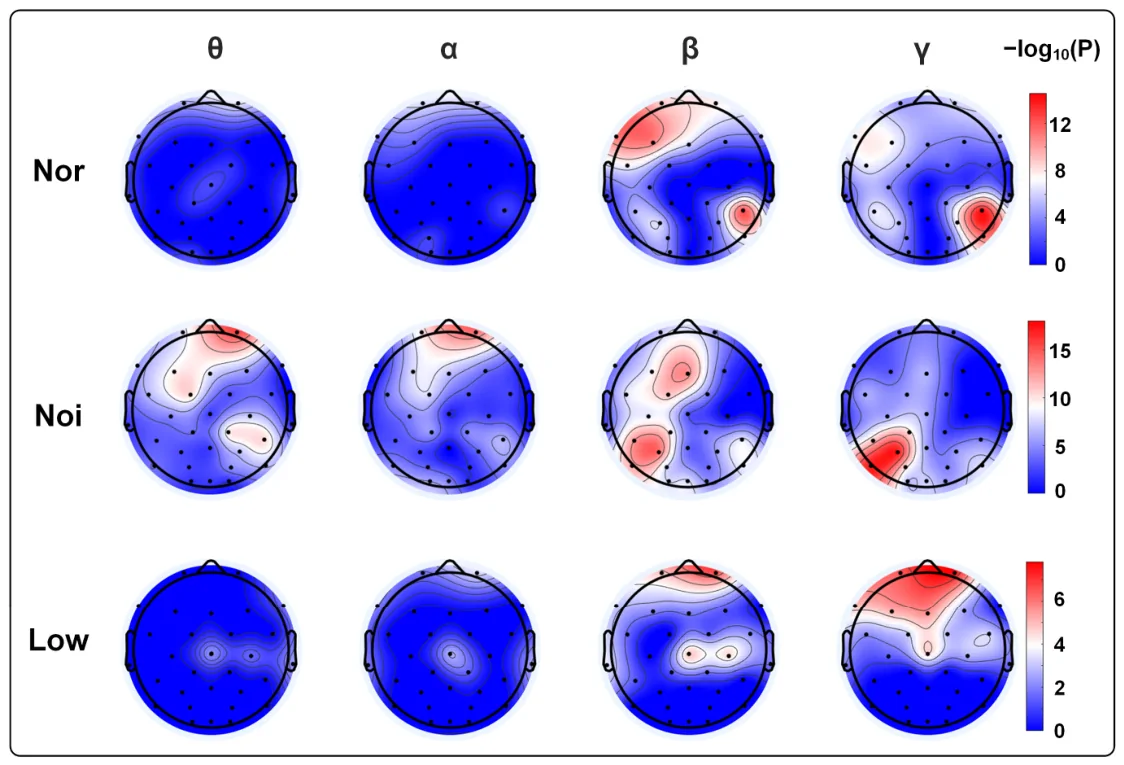
Statistical differences in shooting performance based on band power features across frequency bands under the three conditions (Nor = normal, Noi = noise, Low = low light).

**Figure A3:**
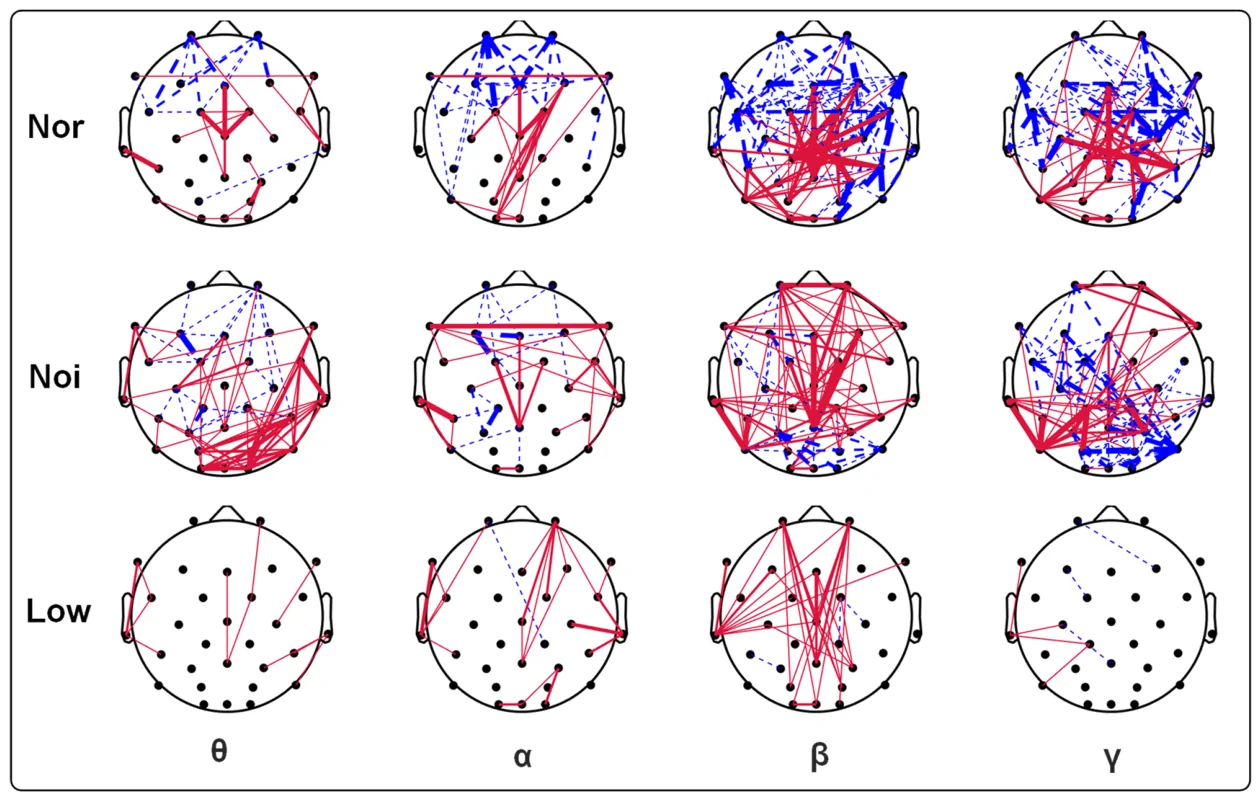
Statistical differences in PLV connectivity features across shooting-performance levels under three conditions (red indicates higher feature values in high-performance groups; blue indicates higher feature values in low-performance groups); (Nor = normal, Noi = noise, Low = low light).

**Figure A4:**
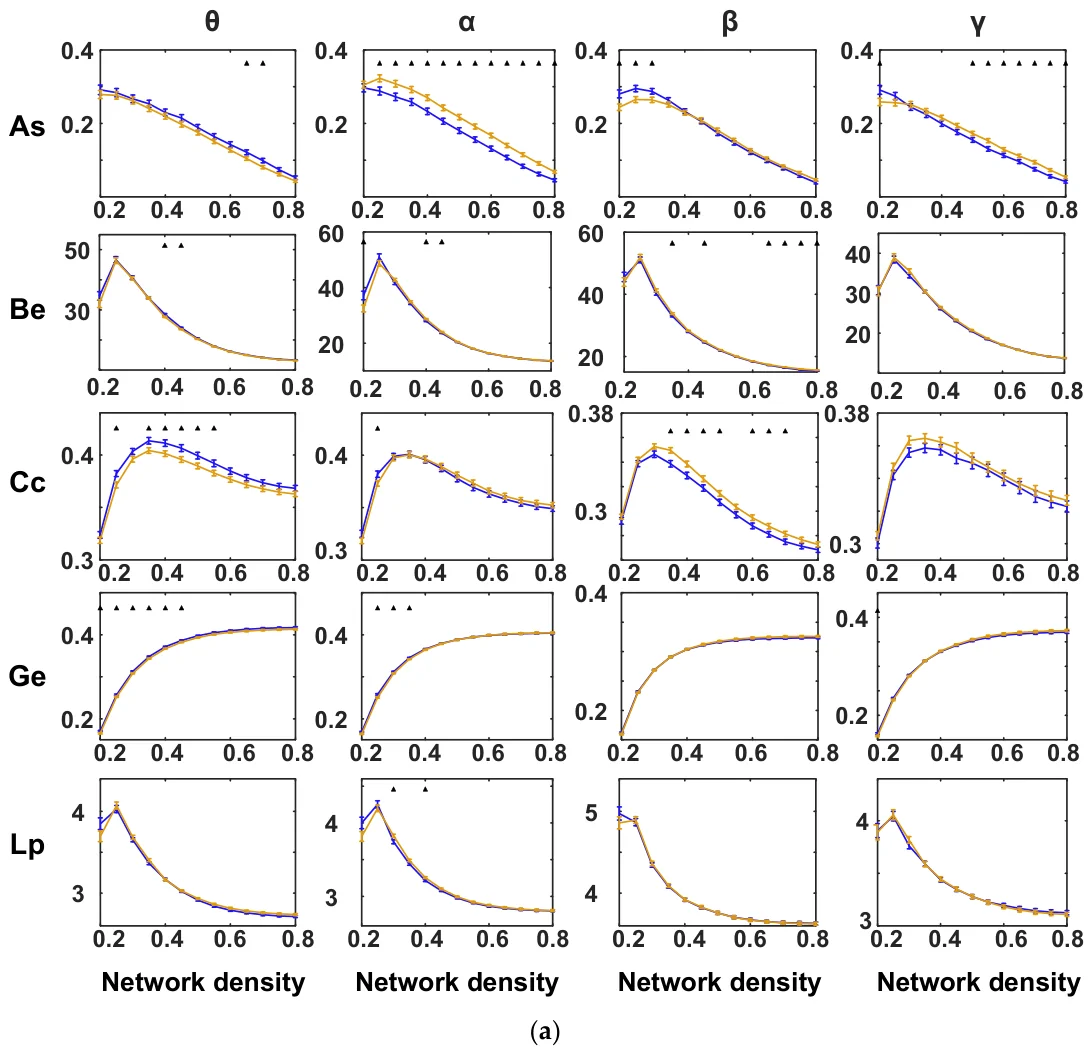

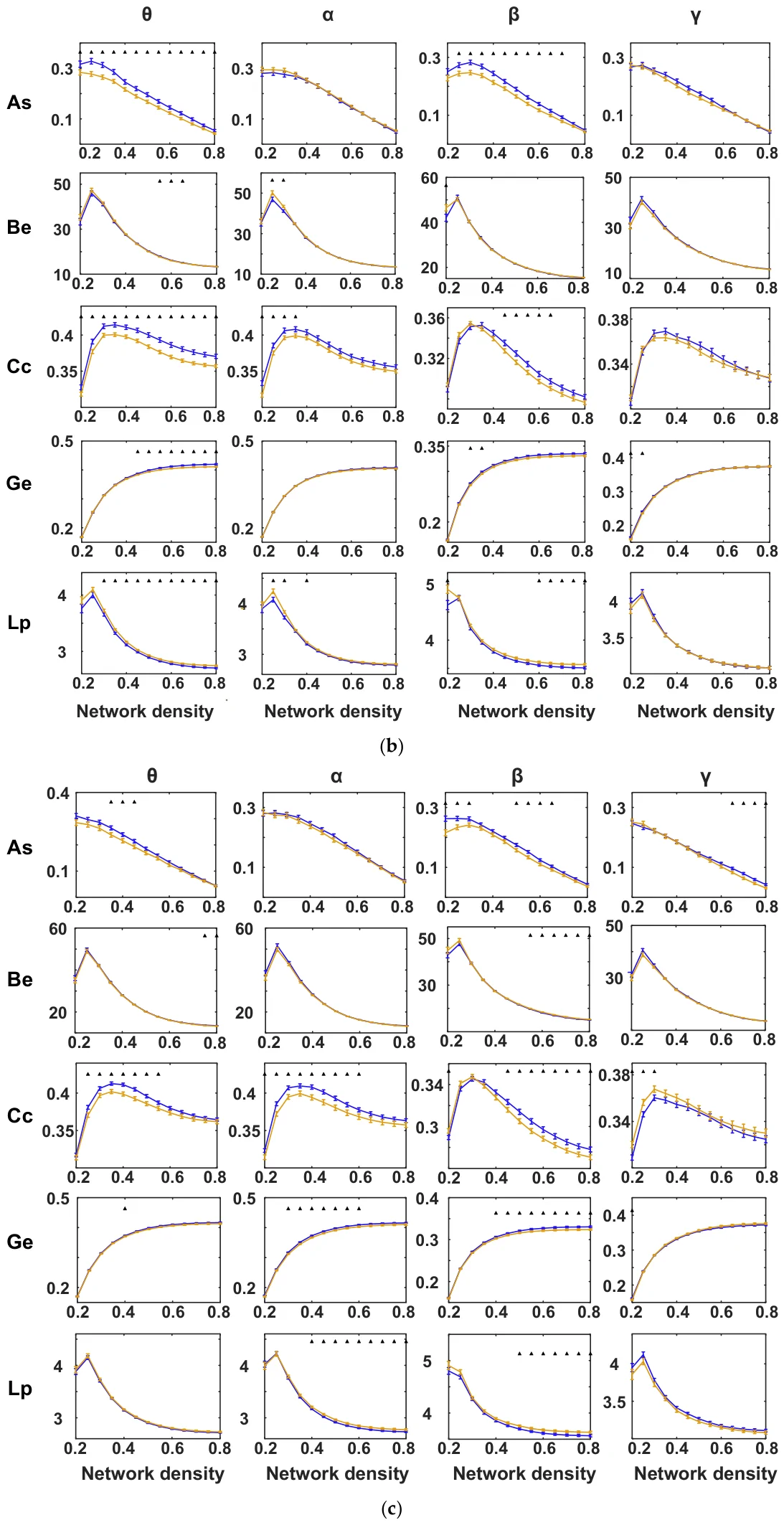
(**a**) Significant differences in brain network features across frequency bands and sparsity levels under the normal condition. (**b**) Significant differences in brain network features across frequency bands and sparsity levels under the noise condition. (**c**) Significant differences in brain network features across frequency bands and sparsity levels under the low-light condition. Blue lines represent the high-performance group, orange lines represent the low-performance group, and black upward triangles (▲) indicate significant differences between groups (*p* < 0.05).

**Figure A5:**
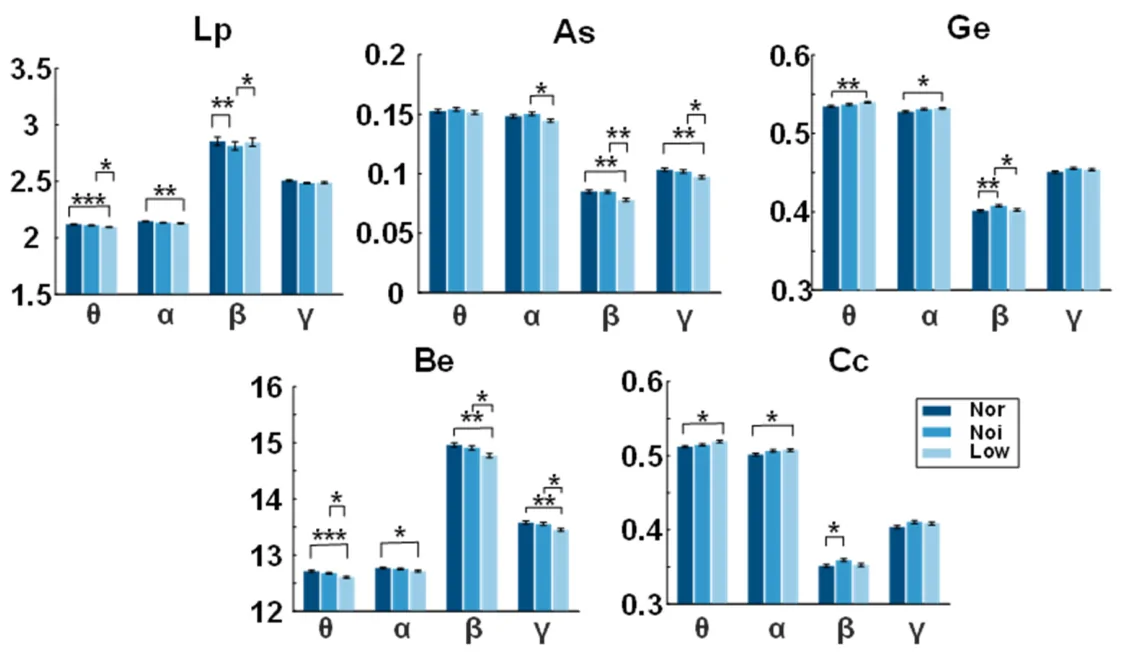
Statistical differences in brain network features across different frequency bands between different conditions (Nor = normal, Noi = noise, Low = low light). * *p* < 0.05, ** *p* < 0.01, *** *p* < 0.001.

**Figure A6:**
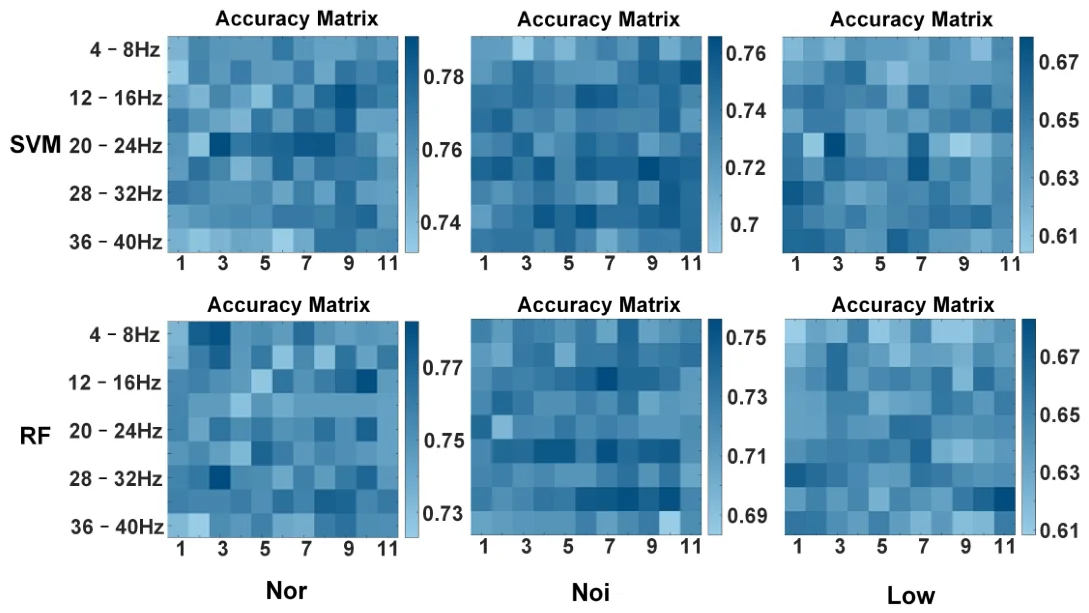
Classification accuracy of FBCSP features across different frequency bands and time windows (Nor = normal, Noi = noise, Low = low light).

**Figure A7:**
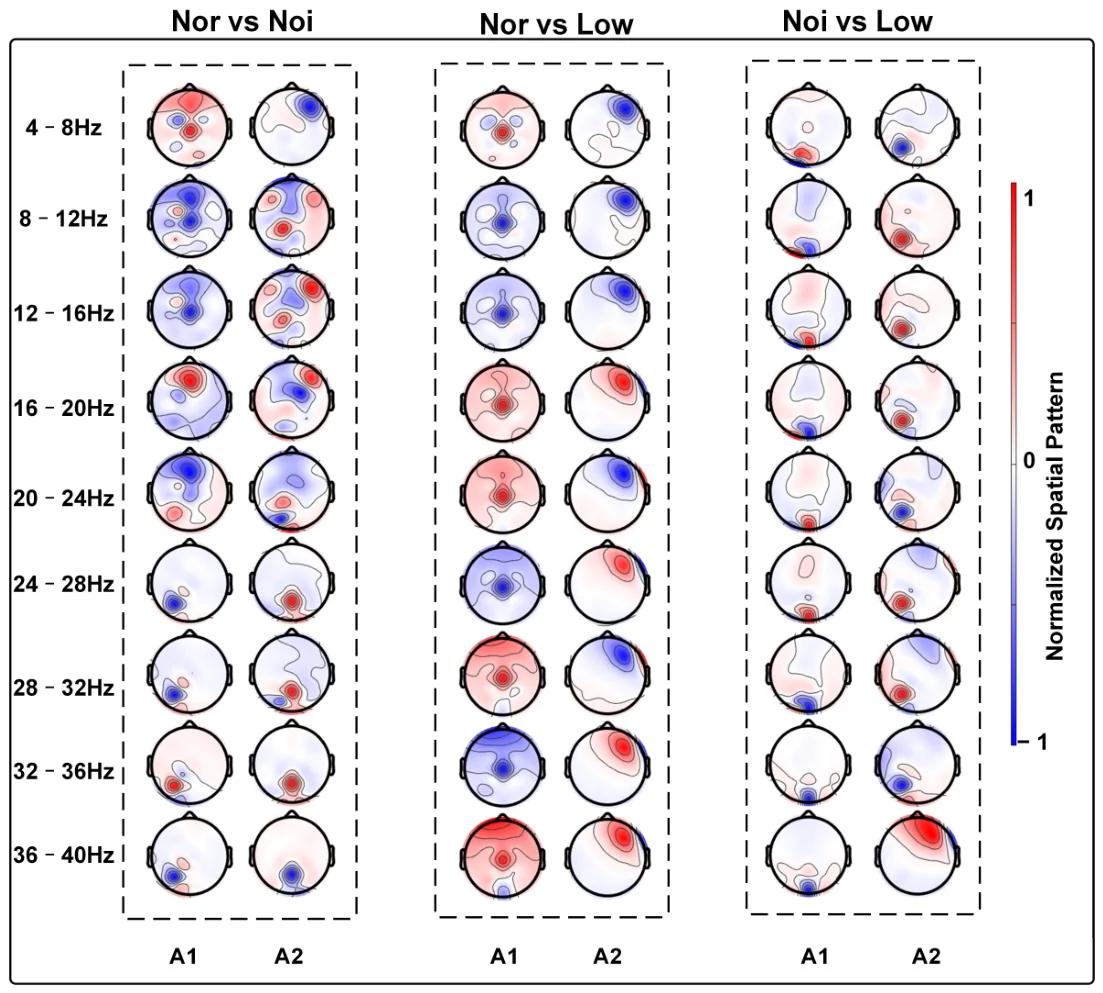
Scalp topographies of spatial filter weights for cross-condition classification (red indicates enhancement, blue indicates suppression) (Nor = normal, Noi = noise, Low = low light).

**Figure A8:**
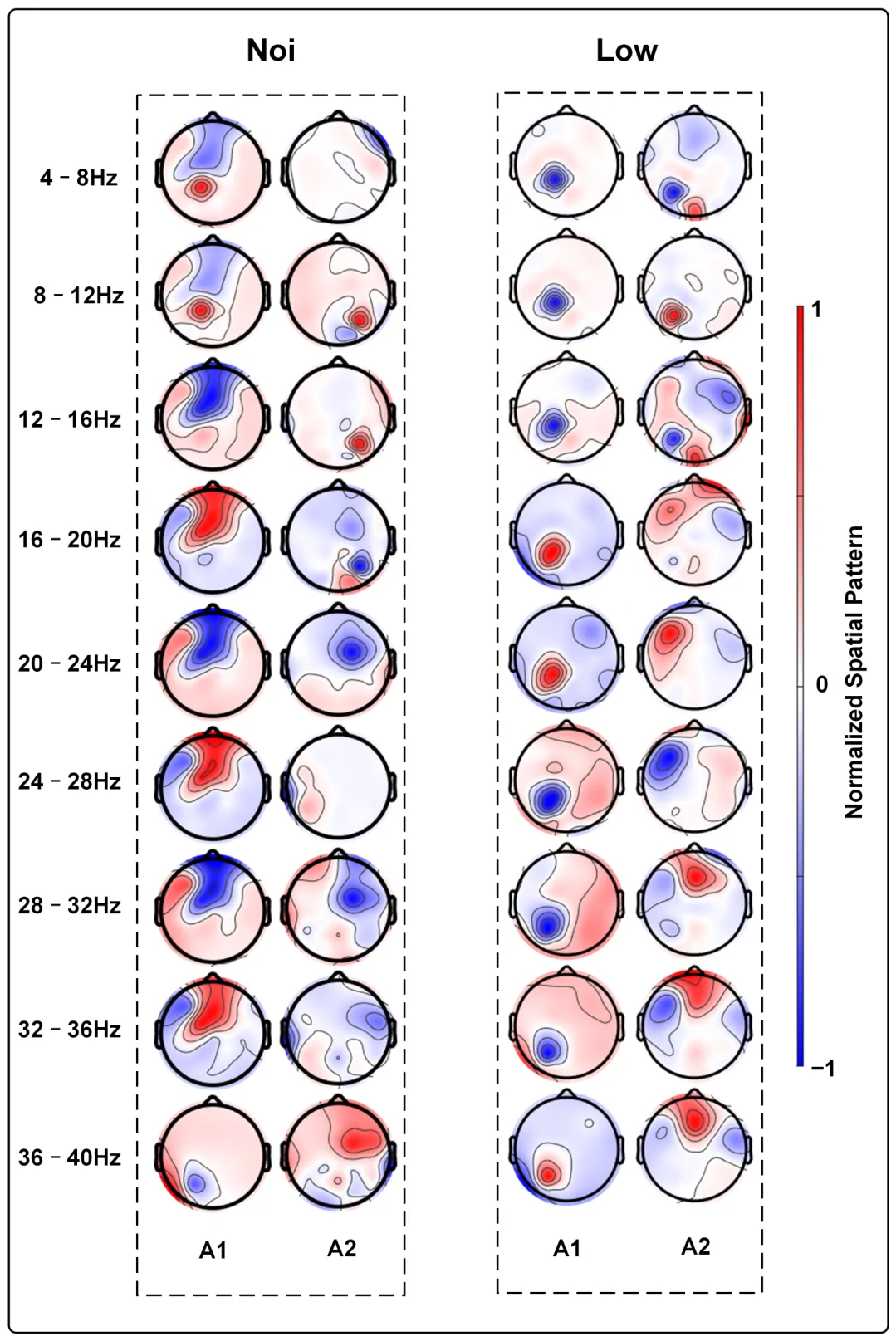
Scalp topographies of spatial filter weights for shooting-performance classification in the noise and low-light conditions (Noi = noise, Low = low light).

**Figure A9:**
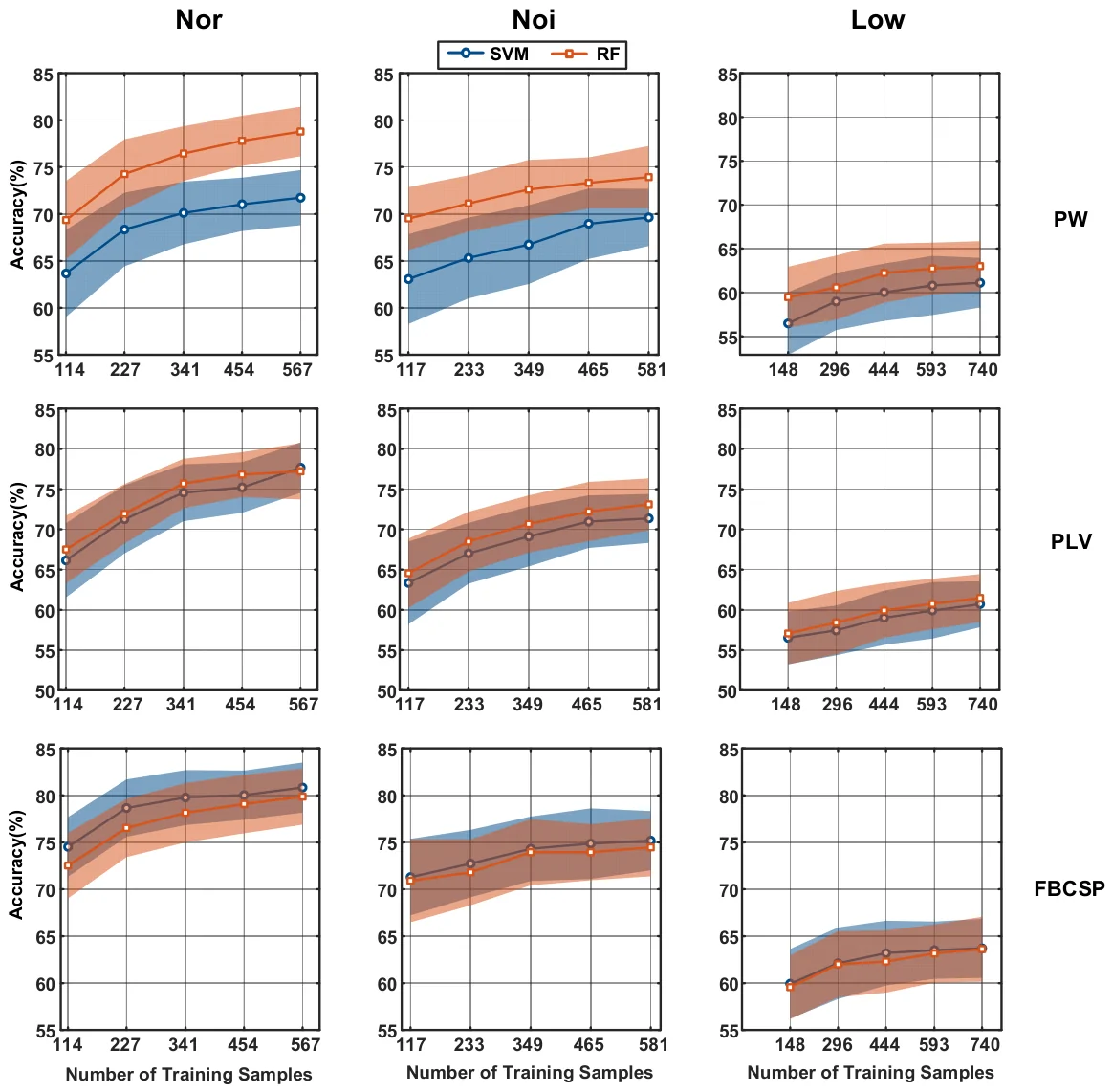
Learning curves of classification models with three feature types across three conditions (different colored lines represent different classifiers as indicated by the legend, and shaded bands indicate the mean ± standard deviation of test accuracy across repeated training runs; Nor = normal, Noi = noise, Low = low light).

**Figure A10:**
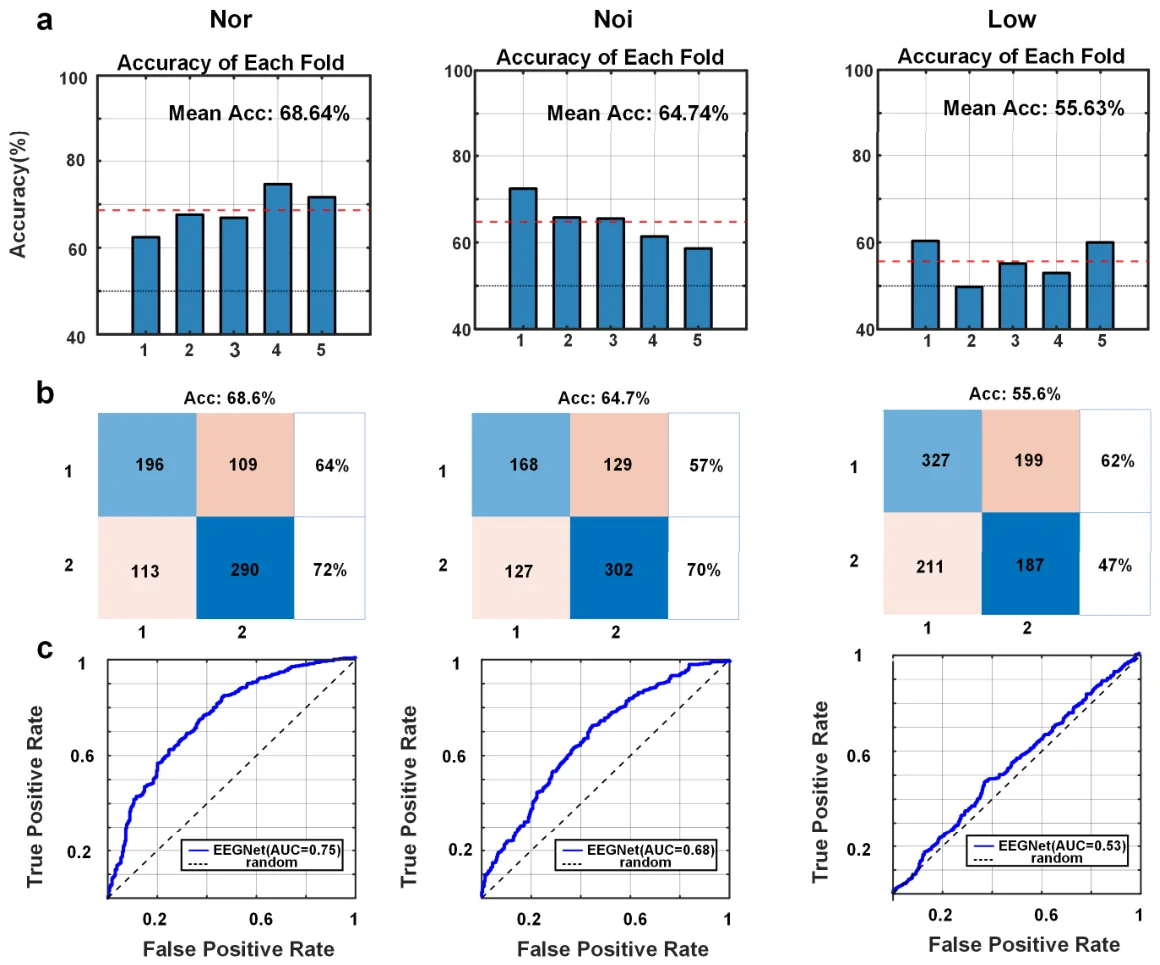
Classification results of shooting-performance EEG using EEGNet across the three conditions. (**a**) five-fold cross-validation classification accuracy; (**b**) confusion matrices; (**c**) ROC curves (Nor = normal, Noi = noise, Low = low light).

## Data Availability

The data presented in this study are available on request from the corresponding author. The data are not publicly available due to privacy concerns related to study participants.

## Appendix A

### Appendix A.1. Statistical Analysis of Power Features and PLV Functional Connectivity Features

Figure A1 shows the inter-subject statistical differences in power features across the three conditions and frequency bands. ANOVA was performed on whole-brain average power across conditions for the four frequency bands to assess statistical differences. The significance level was set at 0.05, with * indicating significance (*p* < 0.05), ** indicating high significance (*p* < 0.01), and *** indicating very high significance (*p* < 0.001). For all statistical analyses, data that did not meet the assumption of normality were transformed using the Box–Cox transformation before testing. As shown in Figure A1, except for the theta band, the low-light condition differed significantly from both the normal and noise conditions. In addition, beta-band power was significantly increased under all three conditions, consistent with previous findings and suggesting that the low-light condition may enhance alertness to some extent.

Figure A2 shows the statistical differences in shooting performance across different frequency bands under the three conditions. For the power features of different shooting performances under the three conditions, Wilcoxon rank-sum tests were conducted across frequency bands and channels, and *p*-values were corrected using the false discovery rate (FDR). For visualization purposes, the results were transformed into negative logarithms (−log(*p*-value)). Red regions in the figure indicate significant differences. Across all three conditions, the beta and gamma bands showed the most significant differences. The statistical test results for the beta band were largely consistent with the channel-contribution patterns of the beta-band RF classification model, providing mutual support and suggesting that the channels playing important roles in classification indeed exhibited significant differences.

Figure A3 shows the statistical differences in PLV-based functional connectivity features across shooting performance levels under the three conditions. For the PLV connectivity features of different shooting performance levels under the three conditions, Wilcoxon rank-sum tests were performed and *p*-values were corrected using the FDR method. Colored connections indicate significant differences (*p* < 0.05), and line thickness indicates the degree of significance. Red lines indicate higher feature values in the high-performance group, whereas blue lines indicate higher feature values in the low-performance group. As shown in the figure, significant differences were observed in the beta and gamma bands. Among the three conditions (normal, noise, and low light), the normal condition showed the largest differences and the low-light condition showed the smallest differences, which is consistent with the classification accuracy trends reported in the main text and further supports the results.

### Appendix A.2. Graph Theory Metrics

This study also investigated the conditional discriminability and shooting-performance discriminability of graph theory metrics derived from brain network connectivity. Global changes in network strength, together with thresholding, can affect network characteristics. In this study, the brain functional network was sparsified into different sparsity levels ranging from 0.20 to 0.80 with a step size of 0.05, and the brain network connectivity features were defined as the feature set across the different sparsity levels.

Based on functional connectivity strength, graph theory metrics characterizing the connectivity patterns of brain functional networks were extracted using the Brain Connectivity Toolbox (BCT; version 2019-03-03) in MATLAB. These metrics included clustering coefficient (Cc), characteristic path length (Lp), global efficiency (Ge), assortativity coefficient (As), and betweenness centrality (Be). Global efficiency measures the efficiency of information transmission across the entire network; characteristic path length represents the average shortest path length between all pairs of nodes in the network; clustering coefficient quantifies the degree of interconnection among a node’s neighbors; betweenness centrality quantifies the proportion of shortest paths between other node pairs that pass through a given node; and assortativity coefficient measures the correlation between a node’s degree and the average degree of its nearest neighbors. In this study, the clustering coefficient refers to the average clustering coefficient, and betweenness centrality refers to the average betweenness centrality.

Table A1 shows the classification accuracy of the SVM model based on brain network graph theory features for different frequency bands across different conditions, while Table A2 presents the classification accuracy of the same model for shooting performance levels across frequency bands under the three conditions. The classification accuracy of the five brain network features, both across different conditions and across different performance levels within the same condition, generally ranged from 0.46 to 0.61, indicating limited discriminative performance.

These results should be interpreted cautiously. Graph-theoretical measures can be sensitive to methodological choices in network construction, including the connectivity estimator, sparsity threshold, and the use of weighted or binarized networks. In addition, global graph measures may not preserve all spatially specific or connection-specific information that is relevant to single-trial EEG classification.

**Table A1:** Classification accuracy of the SVM model based on brain network graph-theoretical features for different frequency bands between different conditions.

		Theta	Alpha	Beta	Gamma
Be	Low vs. Nor	0.53	0.57	0.59	0.58
	Low vs. Noi	0.53	0.56	0.58	0.57
	Nor vs. Noi	0.51	0.46	0.48	0.50
As	Low vs. Nor	0.54	0.53	0.55	0.52
	Low vs. Noi	0.52	0.55	0.54	0.53
	Nor vs. Noi	0.53	0.51	0.53	0.48
Cc	Low vs. Nor	0.53	0.56	0.58	0.55
	Low vs. Noi	0.53	0.53	0.56	0.58
	Nor vs. Noi	0.49	0.54	0.55	0.52
Ge	Low vs. Nor	0.54	0.56	0.57	0.53
	Low vs. Noi	0.53	0.53	0.61	0.57
	Nor vs. Noi	0.51	0.51	0.53	0.51
Lp	Low vs. Nor	0.52	0.55	0.57	0.55
	Low vs. Noi	0.51	0.54	0.55	0.57
	Nor vs. Noi	0.52	0.49	0.51	0.52

(Nor = normal, Noi = noise, Low = low light).

**Table A2:** Classification accuracies of the SVM model based on brain network graph-theoretical features for different shooting performance levels across frequency bands under the three conditions.

		Theta	Alpha	Beta	Gamma
Be	Nor	0.54	0.56	0.56	0.55
	Noi	0.58	0.54	0.59	0.55
	Low	0.51	0.56	0.58	0.54
As	Nor	0.55	0.53	0.54	0.53
	Noi	0.59	0.54	0.56	0.58
	Low	0.53	0.56	0.56	0.53
Cc	Nor	0.56	0.49	0.53	0.56
	Noi	0.59	0.57	0.60	0.55
	Low	0.54	0.54	0.58	0.55
Ge	Nor	0.53	0.56	0.55	0.59
	Noi	0.54	0.58	0.54	0.55
	Low	0.53	0.56	0.53	0.52
Lp	Nor	0.53	0.56	0.55	0.52
	Noi	0.58	0.55	0.54	0.57
	Low	0.55	0.57	0.55	0.50

(Nor = normal, Noi = noise, Low = low light).

Figure A4 shows the statistical differences in various graph theory metrics between shooting performance levels under the three conditions, presenting the mean values and standard errors of the graph-theoretical metrics for the high- and low-performance classes, with *t*-tests performed on the brain network features of the two groups (*p* < 0.05). As shown in Figure A4, under different conditions, various graph theory metrics exhibited significant differences across some sparsity levels and frequency bands. Under the normal condition, significant differences were observed at most sparsity levels for Cc and Ge in the theta band, As in the alpha band, and Be and Cc in the beta band. Under the noise condition, significant differences were found at most sparsity levels for As, Cc, Ge, and Lp in the theta band, and As in the beta band. Under the low-light condition, significant differences were observed at most sparsity levels for Cc in the theta band, Cc, Ge, and Lp in the alpha band, and all five feature groups in the beta band.

Figure A5 shows the ANOVA-based differences in global graph-theoretical features across the three conditions and frequency bands. Global features were obtained by averaging the local features Cc and Be across all channels. Subsequently, ANOVA tests were performed on the global graph-theoretical features under the three conditions across the four frequency bands, with the significance threshold set at 0.05. Specifically, * indicates significance (*p* < 0.05); ** indicates stronger significance (*p* < 0.01); and *** indicates very strong significance (*p* < 0.001). It can be observed that Be, As, and Lp exhibited relatively significant differences across frequency bands, with the low-light condition showing more pronounced differences from the other two conditions.

As shown in Table A1 and Table A2, the classification accuracies of the five brain network features across different conditions and across different performance levels within the same condition were generally around 0.50–0.60, approaching chance level. This may be attributed to several reasons. First, shooting is a task that relies heavily on local network features, whereas global graph-theoretical metrics average the dynamic changes in locally critical nodes with whole-brain background noise during computation, thereby masking spatially specific information. Second, graph-theoretical metrics compress the high-dimensional connectivity matrix into only a few scalar values, resulting in substantial loss of specific connection-path information, in sharp contrast to the favorable classification performance of PLV features. Finally, although Figure A4 and Figure A5 show that graph-theoretical metrics exhibited significant differences in group-level statistical tests, their substantial single-trial variability led to highly overlapping sample distributions, making them unsuitable for single-trial ML classification under the present analysis framework.

Under the present analysis framework, FBCSP and PLV features showed stronger single-trial decoding performance than the examined global graph-theoretical metrics. This finding does not indicate that graph-theoretical measures are inherently unsuitable for EEG classification, because their performance may depend on network construction, feature granularity, and the validation strategy. Future studies should assess the robustness of graph-theoretical measures across alternative connectivity and thresholding strategies and evaluate weighted or threshold-free networks. Incorporating nodal or edge-level graph features, dynamic network measures, or hybrid models combining graph-theoretical features with PLV or spatial features may preserve more discriminative information and improve classification performance.

### Appendix A.3. FBCSP Features

Figure A6 shows the classification accuracy of FBCSP features across different frequency bands and time windows. Under the normal condition, both models achieved better classification accuracy in the 20–24 Hz and 32–36 Hz bands, with higher accuracy observed in the eighth and ninth time windows. Under the noise condition, the two models performed better in the 24–28 Hz and 32–36 Hz bands, with the eighth time window showing higher accuracy. Under the low-light condition, both models achieved better classification accuracy in the 32–36 Hz band, with the seventh time window performing best. Overall, across the 3 s shooting preparation period, classification accuracy was higher in the middle and late time windows.

Figure A7 shows the scalp topographies of spatial pattern weights for cross-condition classification. In the normal vs. noise comparison, the normal condition (A1) showed key discriminative regions in the left parieto-occipital area, whereas the noise condition (A2) showed a shift toward the central region and posterior midline. This may reflect increased perceptual load and cognitive resource reallocation through central multisensory networks to maintain task performance under auditory interference. In the normal vs. low-light comparison, the normal condition (A1) showed a relatively broad central-parietal pattern, whereas the low-light condition (A2) showed a more stable spatial clustering of core weights concentrated in the right frontal/prefrontal region. Previous neurophysiological evidence suggests that the right prefrontal cortex is closely related to sustained attention and vigilance. This pattern may indicate that, under low-light conditions with insufficient visual input, additional top-down compensatory processes are recruited to support visual attention and conditional alertness.

In the noise vs. low-light comparison, the core classification weights were concentrated in the occipital region under both the noise (A1) and low-light (A2) conditions. This suggests that when the classifier directly distinguishes auditory interference from visual restriction, the most informative features may be related to the rhythmic state of visual processing regions rather than broad frontoparietal network differences. Low-light conditions may alter the spatial patterns of the occipital region by reducing visual input, and the classification model may capture these posterior-region changes to distinguish between the two types of cross-modal interference. Only in the 36–40 Hz band did the low-light condition additionally show strong compensatory features in the right frontal region. Overall, the enhanced and suppressed regions differed across condition comparisons, suggesting that different conditions exhibited distinct discriminative neural patterns.

Figure A8 shows the scalp topographies of spatial pattern weights for shooting-performance classification in the noise and low-light conditions. In the noise condition, the high-performance class (A1) showed key discriminative regions in the left prefrontal/frontal cortex, suggesting stronger engagement of executive control processes that may help suppress auditory interference. In contrast, the low-performance class (A2) showed a more dispersed pattern, mainly distributed over posterior or right-sided regions, which may indicate less efficient allocation of attentional resources under noise interference. In the low-light condition, the high-performance class (A1) was mainly concentrated in the left parieto-occipital region, suggesting more effective use of residual visual cortical resources to maintain attention. The low-performance class (A2) was mainly concentrated in the frontal cortex, indicating that visual restriction may require greater frontal compensation for vigilance and control. Overall, the discriminative regions differed across the two conditions, suggesting that high- and low-performance trials are separable and may reflect different neural patterns under different conditional constraints.

### Appendix A.4. Learning Curves

Figure A9 shows the learning curves of SVM and RF classifiers trained with the three feature types across the three conditions. The results indicate that the accuracy of all models increased and then stabilized as the number of training samples increased, supporting the effectiveness of the models.

### Appendix A.5. Shooting Performance Discriminability Based on EEGNet

Figure A10 shows the classification results of shooting-performance EEG based on the EEGNet model across the three conditions. Figure A10a presents the five-fold cross-validation classification accuracy across the following three conditions: the mean classification accuracy was 69% in the normal condition, 65% in the noise condition, and 56% in the low-light condition. Figure A10b shows the confusion matrices across the three conditions; in the normal and noise conditions, specificity was markedly higher than recall. Figure A10c shows the ROC curves across the following three conditions: the AUC was 0.75 in the normal condition, 0.68 in the noise condition, and 0.53 in the low-light condition. Due to the limited number of participants, the use of a basic model configuration, and the absence of extensive architecture or hyperparameter optimization, the classification accuracy was lower than that of the handcrafted-feature pipelines under the present experimental setting. This result suggests that the tested handcrafted-feature pipelines were better suited to the available dataset than the tested EEGNet configuration. It should not be interpreted as evidence that end-to-end deep-learning models are generally inferior for shooting EEG classification.
